# High-throughput screening reveals higher synergistic effect of MEK inhibitor combinations in colon cancer spheroids

**DOI:** 10.1038/s41598-020-68441-0

**Published:** 2020-07-14

**Authors:** Evelina Folkesson, Barbara Niederdorfer, Vu To Nakstad, Liv Thommesen, Geir Klinkenberg, Astrid Lægreid, Åsmund Flobak

**Affiliations:** 1 Department of Clinical and Molecular Medicine, Norwegian University of Science and Technology, Trondheim, Norway; 2 The Cancer Clinic, St Olav’s University Hospital, Trondheim, Norway; 3 Department of Biotechnology, SINTEF Materials and Chemistry, Trondheim, Norway; 4 Department of Biomedical Laboratory Science, Norwegian University of Science and Technology, Trondheim, Norway

**Keywords:** Biological techniques, Cancer, Drug discovery, Medical research, Molecular medicine, Oncology

## Abstract

Drug combinations have been proposed to combat drug resistance, but putative treatments are challenged by low bench-to-bed translational efficiency. To explore the effect of cell culture format and readout methods on identification of synergistic drug combinations in vitro, we studied response to 21 clinically relevant drug combinations in standard planar (2D) layouts and physiologically more relevant spheroid (3D) cultures of HCT-116, HT-29 and SW-620 cells. By assessing changes in viability, confluency and spheroid size, we were able to identify readout- and culture format-independent synergies, as well as synergies specific to either culture format or readout method. In particular, we found that spheroids, compared to 2D cultures, were generally both more sensitive and showed greater synergistic response to combinations involving a MEK inhibitor. These results further shed light on the importance of including more complex culture models in order to increase the efficiency of drug discovery pipelines.

## Introduction

Colorectal cancer (CRC) is the third most common neoplastic malignancy worldwide^1^, and although improvements in standard treatments have increased the survival rates over the past 20 years^2^, far from all patients benefit from currently available therapies. Targeted therapy, using drugs aimed to target specific molecules involved in tumour growth, is being regarded as a promising tool to increase response rates to cancer therapy. However, the number of such therapies that have made it all the way to the clinic has been limited. This may be explained by lack of therapy response due to adaptive drug resistance, or transient response due to acquired resistance. Drug combinations are being discussed as a promising strategy to overcome the resistance frequently observed upon administration of targeted monotherapy^3,4^. The augmented effect of targeted drug combination treatment is frequently ascribed to the drugs’ ability to jointly interfere with the growth-promoting signalling network of cancer cells at multiple points. High-throughput cell line screening platforms have been successfully employed as tools to uncover novel synergistic drug combinations. In the study ALMANAC of the National Cancer Institute (NCI), where a large number of pairwise combinations of FDA-approved cancer drugs were screened in vitro, several novel pairs of synergistic drug combinations were identified, whereof roughly a third also were shown to be efficient and synergistic in vivo^5^. Another example is the Merck Research Laboratories screen, in which 583 combinations of experimental and approved cancer drugs were screened in a panel of cancer cell lines, identifying well-known as well as novel synergistic drug combinations in vitro^6^.

Despite large combination screening efforts with successful hits in vitro, putative treatments are challenged by low bench-to-bed translational efficiency. The insufficient ability of cell lines grown on planar surfaces to correctly recapitulate drug response in vivo has been debated as a possible explanation for this^7^. Accompanied by several studies pointing towards signalling and response differences between planar (2D) and spheroid (3D) cultures in vitro^8,9^, it has been discussed whether spheroid cultures would offer a more reliable in vitro system. Although different cultivation techniques allow for different levels of complexity of 3D cultures^7^, they all share the common characteristic of representing a cellular architecture with physiologically relevant gradients, not present in planar cultured cells^8,10^. These gradients relate to e.g. concentrations of nutrients, growth factors, oxygen and drugs, which have been shown to mimic corresponding gradients in patient tumours, including chemical gradients set up by the proximity of blood vessels in vivo^8^. In contrast to 2D-cultured cells, where the larger part of the cell population is actively proliferating, 3D cultures are considerably more heterogeneous with respect to the proliferative capacity and have, unlike cells cultured in 2D, been found to contain a non-proliferating quiescent or hypoxic cell population similar to that of tumours in vivo^8^. Clinically, quiescent tumour cell populations constitute a major treatment hurdle, as the quiescent phenotype frequently is associated with resistance to standard therapies^11,12^. Monitoring the effect of drugs considering also non-proliferating cells may therefore be of great significance in order to increase the bench-to-bed translational efficiency. Overall, these considerations are some, among many others, that may partly explain why drugs with documented efficiency in 2D cultures often do not show the same effect in more complex cellular contexts and in vivo.

In the present study, we have performed a high-throughput screen to systematically compare drug combination effects in 2D versus 3D culture models of three CRC cell lines (HCT-116, HT-29 and SW-620). The combinatorial treatments investigated comprised all pairwise combinations of five experimental or approved targeted small molecule inhibitors and two approved chemotherapeutic drugs. Our results show that several drug combination effects are observed in only one of the culture modes as measured by ATP content, a widely used readout for cell viability. Inclusion of cell confluency and spheroid size as additional cell growth readouts identified additional synergistic combinations, although synergistic drug combinations called by the different readouts overall showed high agreement within culture formats. These findings highlight the importance of more advanced screening platforms, encompassing different phenotypic readouts and more so, 3D culture models, for identification of synergistic drug combinations.

## Results

### Screening procedure

**Figure 1:**
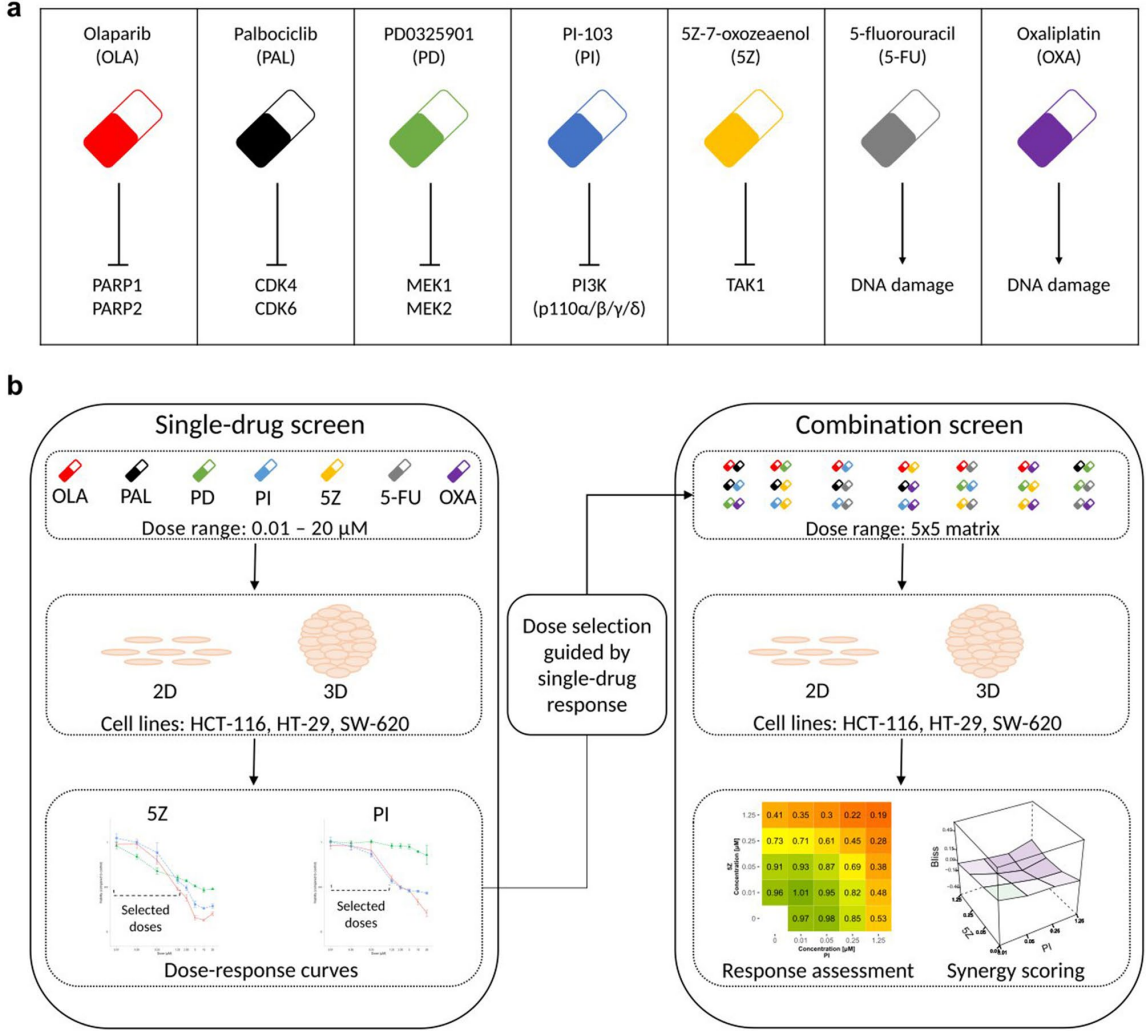
Overview of drugs, targets and screening procedure. (**a**) Drugs included in the study are presented with their full name, abbreviation and target/effect. (**b**) Single-drug screen: cells were treated with each drug in single application in a broad dose range. Combination screen: drugs were combined pairwise in 5 × 5 matrices. Dose selection was guided by single-drug response. In both screens, cells were subjected to drugs or drug combinations for 48 h. Combination effect was calculated using the Bliss independence reference model. Responses in both single and combination screens were assessed by measuring cell viability (ATP-content, CellTiter-Glo). Cell confluency and spheroid size was additionally quantified in 2D- and 3D-cultured cells, respectively.

To identify efficacious synergistic drug combinations, we screened five targeted and two chemotherapeutic drugs in 2D and 3D CRC cell line cultures (HCT-116, HT-29, SW-620). Drugs were selected based on approval for clinical use in CRC or other cancer types (5-FU, oxaliplatin, olaparib, palbociclib), and on their ability to target pathways frequently dysregulated in cancer (MAPK/ERK pathway, PI3K/AKT/mTOR pathway and TGF-beta pathway). The combination screen, in which all 21 pairwise combinations were screened in 5 × 5 dose matrices, was preceded by a single-drug screen, where cells were subjected to a broad dose range (0.01–20 µM) of the drugs in single application. Results from the single-drug screen were used to guide the selection of doses for the combination screen (Fig. 1). In line with procedures applied by other drug screen labs^5,6,13,14^, we used viability as assessed by ATP content (CellTiter-Glo) as the main readout to gauge drug responses in 2D and 3D cultures. Additional readouts included measurement of confluency (2D), spheroid diameter (3D) and cell death (2D).

### MEK and TAK1 inhibitors most strongly compromise cell viability upon single-drug treatment

**Figure 2:**
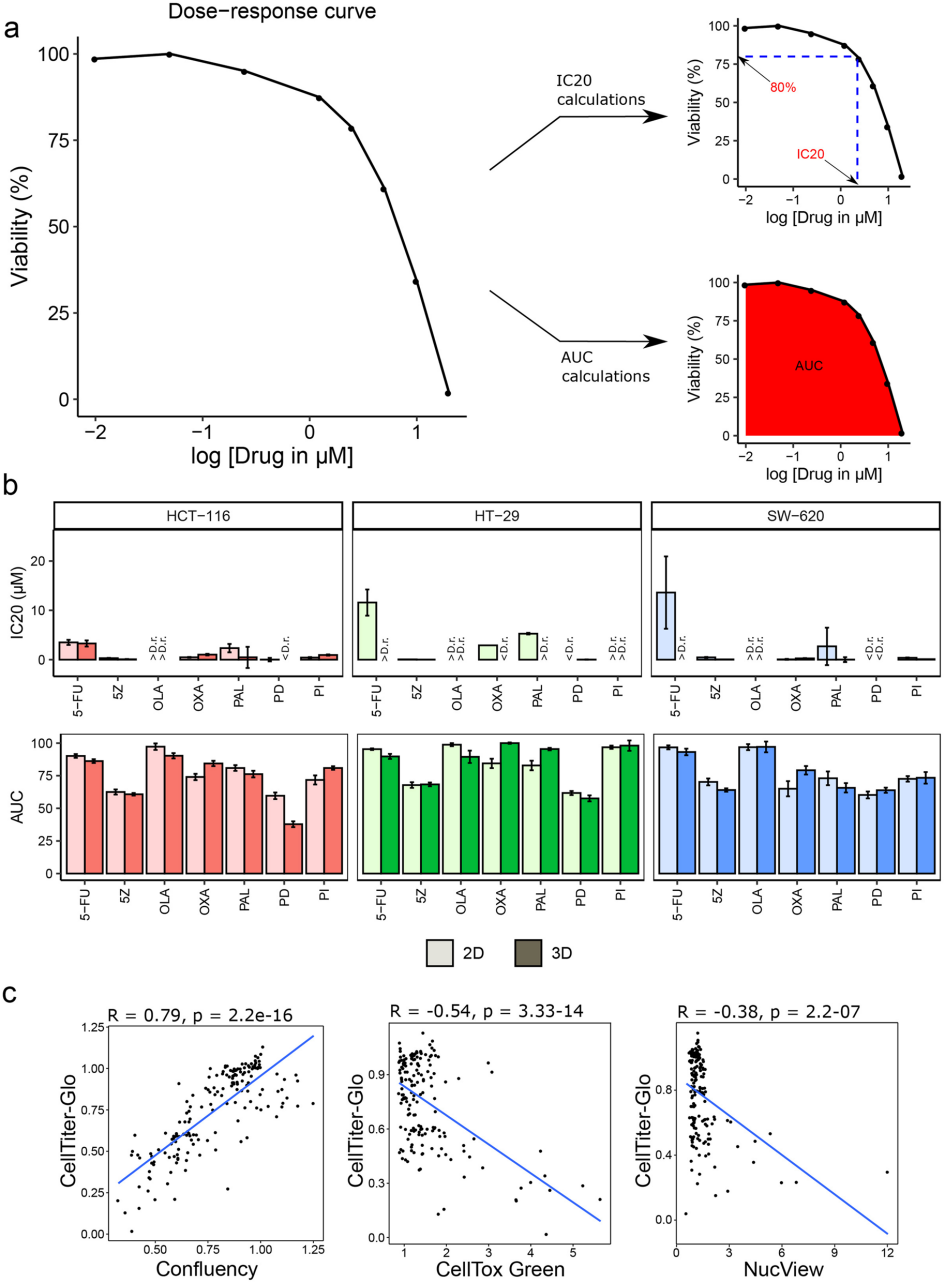
Single-drug screen data. (**a**) Principle: IC20 and AUC values were estimated from viability-based dose–response curves per cell line, culture format and drug. (**b**) Bar plots of IC20 and AUC values, where < D.r. and > D.r. indicate values below and above the tested dose range (0.01–20 µM), respectively. NA indicates that no IC20 could be calculated. Error bars represent standard error of the mean (SEM) of four technical replicates. (**c**) Correlation between CellTiter-Glo (viability) and other responses (confluency, CellTox Green and NucView) in 2D cultures following 48 h of incubation with single-drugs.

To evaluate the optimal dose range for the drug combination screen, we performed curve fitting^15^ and calculated IC20 and Area Under the Curve (AUC) values (Fig. 2a,b) based on single-drug response viability data (Supplementary Table S1, Supplementary Fig. S4). As shown in Fig. 2a,b, the MEK (MAP2K1, MAP2K2) inhibitor PD0325901 (PD) was found to be the most potent single-inhibitor across all cell lines in both 2D- and 3D-cultured cells, followed by the TAK1 (MAP3K7) inhibitor 5Z-7-oxozeaenol (5Z). Comparison of drug responses between culture formats (2D versus 3D), indicated that HT-29 cells were less sensitive to oxaliplatin (OXA) and palbociclib (PAL) when cultured in 3D, while HCT-116 appeared to be more sensitive to MEK inhibition in the 3D format, compared to planar cultured cells. Although comparison between 2D and 3D cultures revealed general response differences between the two culture formats, no clear trend pointing towards either of them being more sensitive than the other was observed.

### Single-drug treatment reduces viability independently of cell death

As the CellTiter-Glo ATP assay provides viability information based on cellular metabolic activity^16^ rather than giving an absolute phenotypic outcome, we additionally assessed confluency and cell death^17,18^ in 2D-cultured cells and included assessment of spheroid size in 3D-cultured cells^19,20^. Although most drugs showed effect in terms of reduced viability, which was also accompanied by a reduction in relative confluency (Supplementary Figs. S4, S5), increased levels of caspase-3 (NucView) or cellular DNA (CellTox Green) were rarely observed for any of the single-drugs (Supplementary Fig. S6). Out of the seven single-drugs, only the TAK1 inhibitor (5Z) induced cell death detectable by both cell death assays at several concentrations across all cell lines. Apoptotic effects of the TAK1 inhibitor have previously been reported in HeLa and HT-29 cells, where TAK1 inhibition using 5Z-7-oxozeaenol was found to downregulate the apoptosis inhibitor NF-κB in a dose-dependent manner^21^. The overall little effect of single drugs on cell death was also reflected in considerably stronger correlation between cell viability and cell confluency responses compared to the correlation between the viability readout and either of the cell death readouts (Fig. 2c). While none of the treatments reduced confluency compared to start of treatment (Supplementary Fig. S5a), several of the compounds reduced confluency relative to untreated cells upon 48 h exposure (Supplementary Fig. S5b), indicating a cytostatic rather than cytotoxic effect. Spheroid size reported an overall response similar to ATP, with a correlation coefficient of R = 0.73, and was found to be only weakly affected by treatment, with the TAK1 inhibitor having the largest effect followed by the MEK inhibitor (Supplementary Fig. S7).

In summary, our results suggest that, at least in 2D cultures, most of the tested single-drugs reduce viability independently of apoptosis. Overall, response in 3D correlated well with 2D response. As only two of the tested compounds induced cell death at doses selected for the combination screen, this readout was omitted in the combination screen.

### Synergistic drug combinations are more frequently observed in 2D cultures

Next, the drugs were combined in all possible pairwise combinations across all doses in a 5 × 5 matrix (Supplementary Methods: Table II). Drug combination effects were evaluated using the Bliss independence model^22^, where Bliss excess values below and above 0 were classified as synergy and antagonism, respectively. The choice of the Bliss independence model as synergy metric was based on that it is, alongside Loewe additivity and the extension of Combination Indexes, one of the most widely used synergy metrics^23,24^.

**Figure 3:**
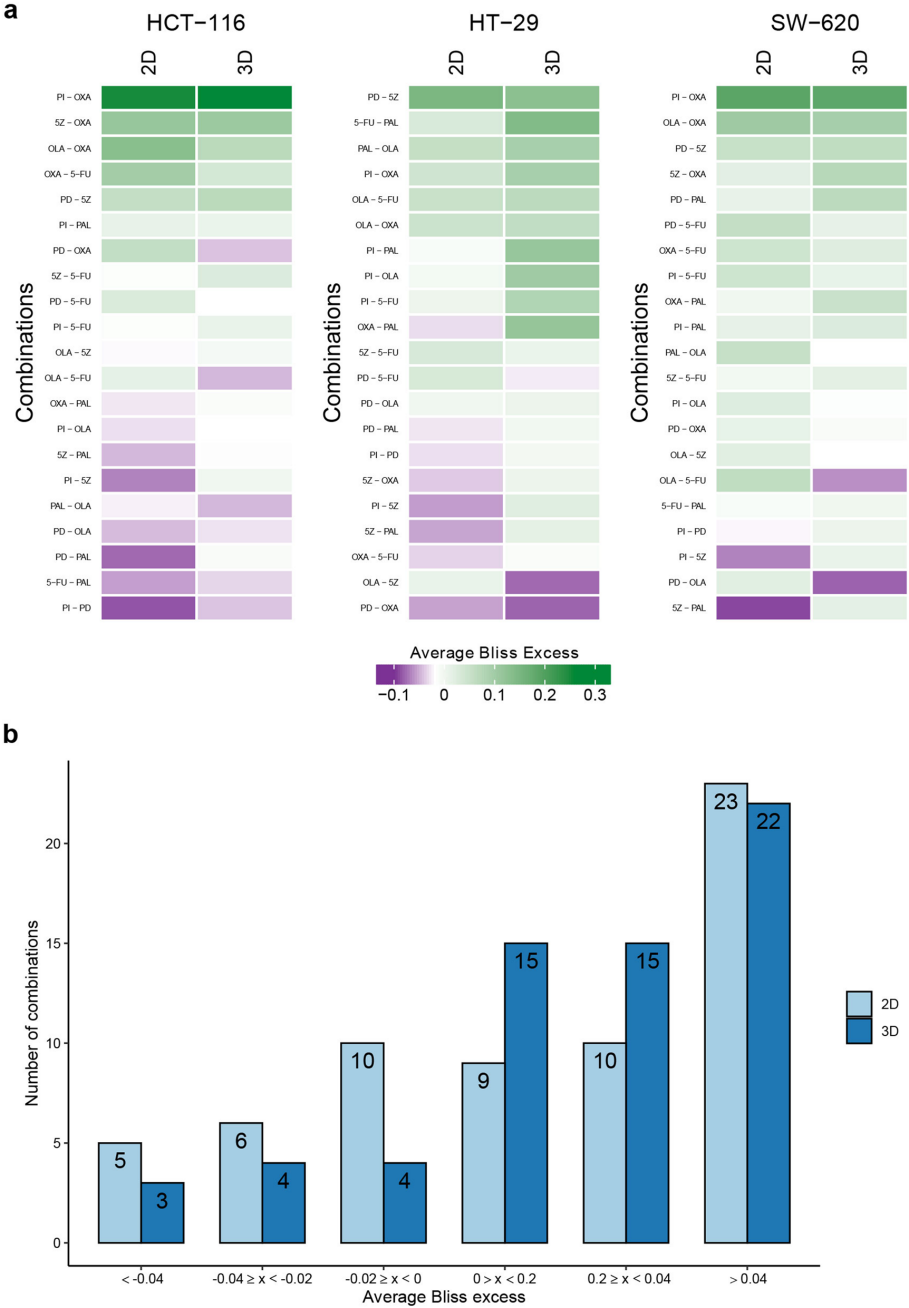
Drug combination effects of tested compounds in 2D and 3D cultured cell lines. (**a**) Heatmaps of Bliss excess averaged across the matrix per combination, cell line and culture format, within cell line comparison of 2D and 3D cultures. Rows are sorted based on Euclidean distance. (**b**) Number of drug combinations showing Bliss excess within given intervals. Combinations with Bliss excess < 0 are classified as synergistic.

Out of all tested combinations in both 2D- and 3D-cultured cells, we observed that approximately 36% (369 of 1,008 data points, 2D) and 35% (351 of 1,008 data points, 3D) showed a greater than expected combination response (viability), i.e. Bliss excess < 0. Of the 21 pairwise drug combinations, 13 and 8 further showed an average Bliss excess < 0 across the whole dose–response matrix in at least one cell line in 2D and 3D, respectively (Fig. 3a). The combinations found to be synergistic included the well-documented combination effect of co-targeting PI3K and MEK^25–27^ as well as combined application of the PI3K inhibitor with the TAK1 inhibitor, previously reported by us^28^ and later also by others^29^. The clinically approved combination of oxaliplatin (OXA) with 5-fluorouracil (5-FU)^30^ was found to be synergistic at low doses of oxaliplatin across all cell lines, albeit with low efficacy of only reducing viability to < 0.5 in 2D-cultured HCT-116 cells. Other drug combinations deemed to be synergistic include palbociclib with either the TAK1 inhibitor, oxaliplatin and the MEK inhibitor (Fig. 3). As can be seen in Fig. 3b, in general fewer combinations were observed to be synergistic when cells were assayed in 3D as compared to 2D. While in 2D, six combinations were identified to be synergistic in more than one cell line, in 3D, only three combinations—olaparib with 5-FU, and the MEK inhibitor with either of olaparib and oxaliplatin, displayed synergistic action in more than one cell line. This may indicate that cell line-dependency of combination effects is more pronounced in 3D or may be attributed to a generally lower overall number of synergistic combinations in 3D. Interestingly, none of the combinations identified as synergistic in more than one cell line in 3D were among the combinations identified as synergistic in more than one cell line in 2D-cultured cells.

Together, these results indicate that not only is cell line dependency of drug combination effects more pronounced in 3D, but it is even more profoundly different. In summary, our findings indicate that the drug combination effects vary depending on whether planar or spheroid cultures are studied, and that frequently, for a given cell line, one specific combination can be found to act synergistically in only one of the culture formats.

### Synergy-viability plots identify MEK inhibitor combinations as more synergistically effective in 3D cultures

**Figure 4:**
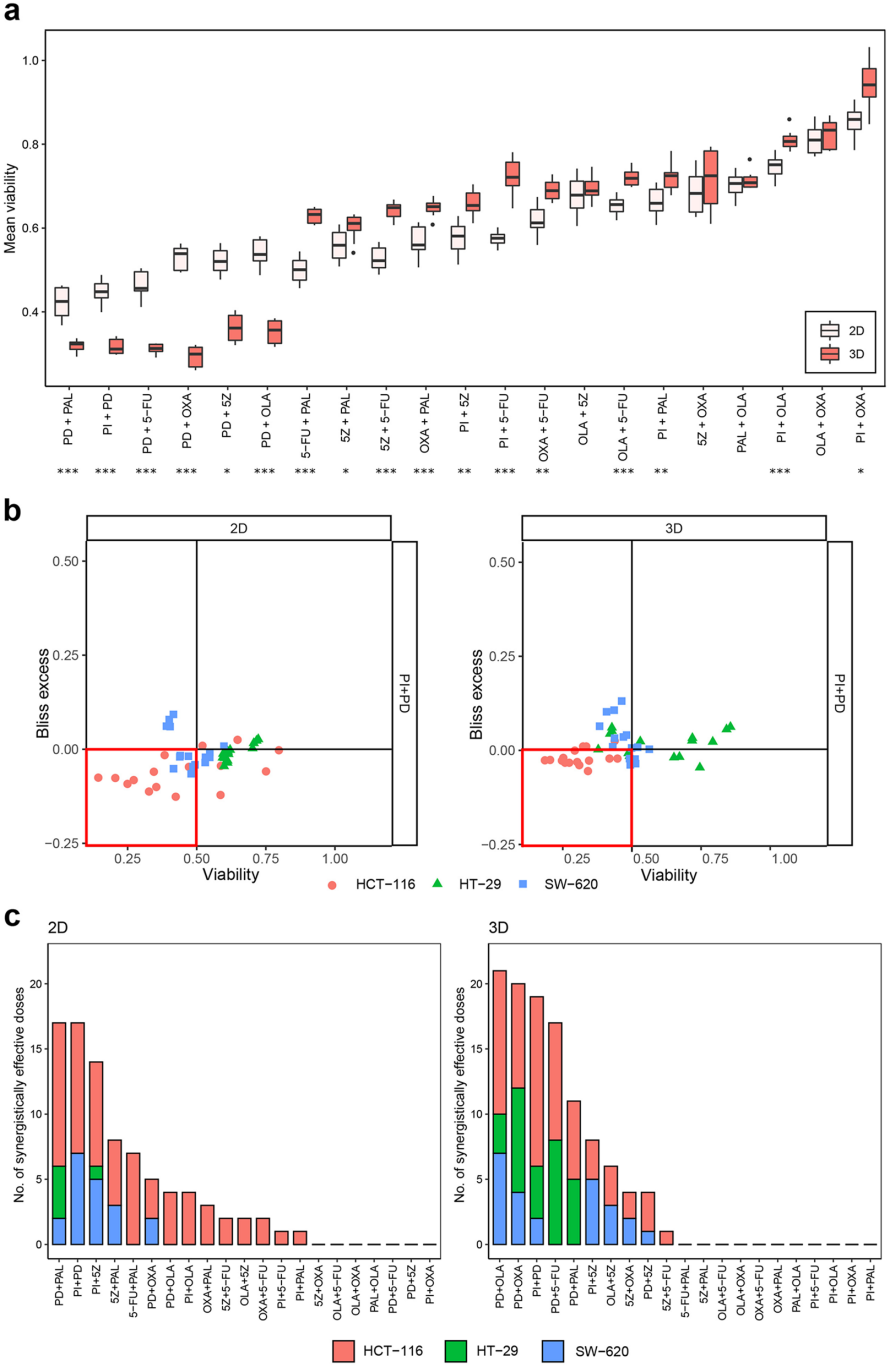
Drug combination effects judged by combined synergy-viability assessments. (**a**) HCT-116 viability averaged across the matrix per drug combination and culture format (2D, 3D). Asterisks (*) indicate a statistically significant difference in average viability between 2D- and 3D-cultured cells per drug combination, with p ≤ 0.05, p ≤ 0.01 and p ≤ 0.001 for *, ** and ***, respectively. (**b**) Bliss excess versus viability plots for 2D- and 3D-cultured cells treated with PI3K inhibitor and MEK inhibitor (PI + PD). Red boxes enclose data points considered to be synergistically effective according to the definition (Bliss excess < 0, viability < 0.5). (**c**) Number of synergistically effective doses per combination, cell line and culture format. Empty positions along the x axis indicate combinations for which no synergistically effective doses were observed (alphabetically per culture format).

Synergy scores give an estimate of the interaction effect of drugs, but do not inform about the magnitude of remaining viability of cells following treatment. Hence, two different combinations might score as equally synergistic, even though both single-drugs and the combination, affect viability considerably more in one pair compared to the other pair and may thus be of higher interest for further characterisation. To take this into account, we introduced two additional measures of combination effects; one by which effect on viability was assessed *without* taking synergy scores into account (effective combination = combination that strongly compromises viability), and one by which combinations were evaluated jointly based on their effect on viability *and* synergistic properties. We use the term ‘synergistically effective combination’ for combinations that act synergistically *and* strongly compromise viability (i.e. ≤ 50% for one or several doses). To evaluate the absolute combination treatment effect on viability, we averaged viability data over the whole matrix per cell line and drug combination. We found that combinations involving the MEK inhibitor most strongly reduced this viability score in both 2D- and 3D-cultured cells, with significantly increased sensitivity in 3D compared to 2D for several combinations (Fig. 4a, Supplementary Fig. S8). Although 2D cultures were generally found to be more sensitive when assessed across all drug combinations, 3D cultures tended to be more sensitive to combinations involving the MEK inhibitor. For HCT-116 the higher sensitivity of spheroids was significant for all combinations involving the MEK inhibitor, whereas in HT-29 and SW-620 cells, it was evident for three and two combinations, respectively (Fig. 4a, Supplementary Fig. S8). Although strongly effective in both culture formats, the synergistic effect of MEK inhibitor combinations, compared to non-MEK inhibitor combinations, was generally weaker in 2D compared to 3D (Fig. 3a). This was also reflected in number of ‘synergistically effective combination’ concentrations (Fig. 4b,c). Here, five out of six MEK inhibitor combinations were among the most synergistically effective combinations in 3D, whereas only two of these combinations were among the five most synergistic and effective combinations in 2D (Fig. 4c). These results indicate that whereas in 2D cultures high sensitivity towards MEK inhibition alone most likely accounts for the strong reduction in viability observed upon treatment with MEK inhibitor combinations, the viability reduction in 3D cultures is a synergistic effect that can to a larger extent be ascribed to both drugs in the pairwise combinations involving the MEK inhibitor. Overall, the landscape of synergistically effective combinations appears to be more diverse in 2D cultures, with four different drugs (PD, PAL, PI and 5Z) involved more than once in the top five combinations (Fig. 4c), compared to only two different drugs (PD and PI) in 3D cultures.

In summary, by implementing the definition ‘synergistically effective combinations’ we were able to identify drug combinations with viability-compromising, as well as synergistic, properties. This strategy further allowed us to identify MEK inhibitor combinations as more synergistically effective in 3D compared to 2D cultures. Several of the combinations classified as synergistically effective have been tested in clinical trials (including the MEK inhibitor with either PI3K inhibitor or palbociclib), alluding the potential clinical value of this scoring metric.

### Synergistic combinations show high agreement within culture formats

**Figure 5:**
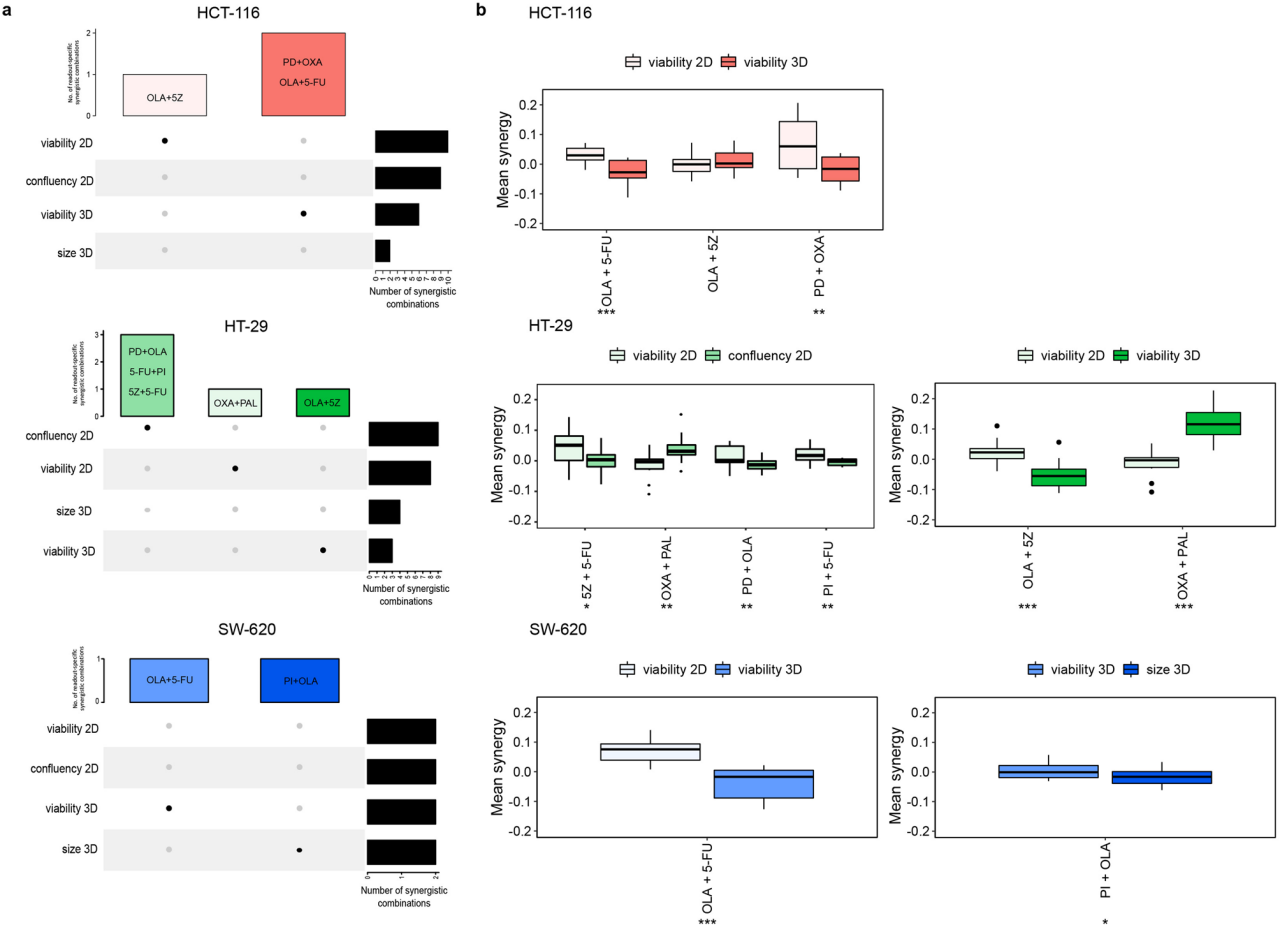
Differences in synergy calling per readout. (**a**) Total number of synergistic drug combinations called per readout (black bars), and total number of readout-specific synergistic combinations (coloured bars), where filled data-points highlight the readout by which synergistic drug combination(s) shown in the coloured bars are uniquely called. (**b**) Differences in synergy strength between indicated readouts per cell line. Asterisks (*) indicate a statistically significant difference in synergy strength, with p ≤ 0.05, p ≤ 0.01 and p ≤ 0.001 for *, ** and ***, respectively.

After studying differences in drug combination response between 2D and 3D-cultured cells by standard viability readout, we further investigated whether imaging-based readouts can provide us with additional distinct information regarding the combinatorial effect of drugs. For this we studied overall Bliss excess scores for synergy classification per combination, cell line and readout. None of the observed synergies were called based on data from all readouts across all cell lines and in common between both 2D- and 3D-cultured cells (Supplementary Fig. S9, Supplementary Table S2). In 2D-cultured HCT-116 and SW-620 cells, synergistic combinations identified by confluency were also identified as synergistic based on viability, while synergy by viability did not necessarily imply synergy by confluency (Supplementary Table S2). In HT-29 cells three combinations were classified as synergistic based on confluency but not by any other readout (Fig. 5a, Supplementary Fig. S9). These combinations showed significantly stronger synergistic response when assessed by confluency compared to the viability readout (Fig. 5b), however, none of the combinations showed strong effect on growth inhibition (Additional file 5). While the largest number of synergistic combinations was called by viability and confluency readouts of 2D-cultured cells, additional distinct synergistic combinations were captured by the two different 3D readouts (Fig. 5a). In total four combinations were only observed using the 3D viability readout (Fig. 5a), out of which one combination (PD + OXA in HCT-116) showed synergistically effective doses within the tested dose range (Fig. 4c).

These results imply that for 2D-cultured HCT-116 and SW-620 cells there is a strong resemblance in the synergistic landscape uncovered by confluency measures compared to the synergies we see based on cell viability (Supplementary Table S2). While in general drug combinations show a lower effect on confluency than on 2D viability (Additional file 1 & 5), combinations found to be synergistically effective in reducing relative viability (Fig. 4c), were also found to be synergistically effective in reducing relative confluency (Supplementary Fig. S10a). The same trend can be observed when comparing synergistic combinations that show an effect on spheroid viability (Fig. 4c) and size (Supplementary Fig. S10b). To summarise, while different readouts within the same culture format overall show high agreement in synergy calling, additional synergistic combinations of potential interest are revealed by screening in 3D cultures, in addition to standard 2D cultures.

### Prolonged drug exposure alters drug combination effects and induces apoptosis upon MEK inhibition

As several drug combinations were found to potently affect viability, we next explored whether also apoptosis was induced and if observed drug effects were reversible or increased with longer exposure time. For this, we continuously monitored apoptosis in addition to cell confluency (2D) and spheroid size (3D) and increased incubation time to 96 h. Viability was included as endpoint measurement for both 2D- and 3D-cultured cells. Three combinations were selected for this follow-up screen based on (1) their synergistic effectiveness (viability ≤ 50% and Bliss excess < 0) in both 2D and 3D at 48 h (5Z + PI, Fig. 4c), or (2) their stronger synergistic effect (Bliss excess) in 3D versus 2D culture at 48 h (PD + OXA, Fig. 3a), or (3) the observation of few synergistic and effective doses across all tested conditions at 48 h (PI + 5-FU, Fig. 4c), but with clinically relevant targets^31,32^.

**Figure 6:**
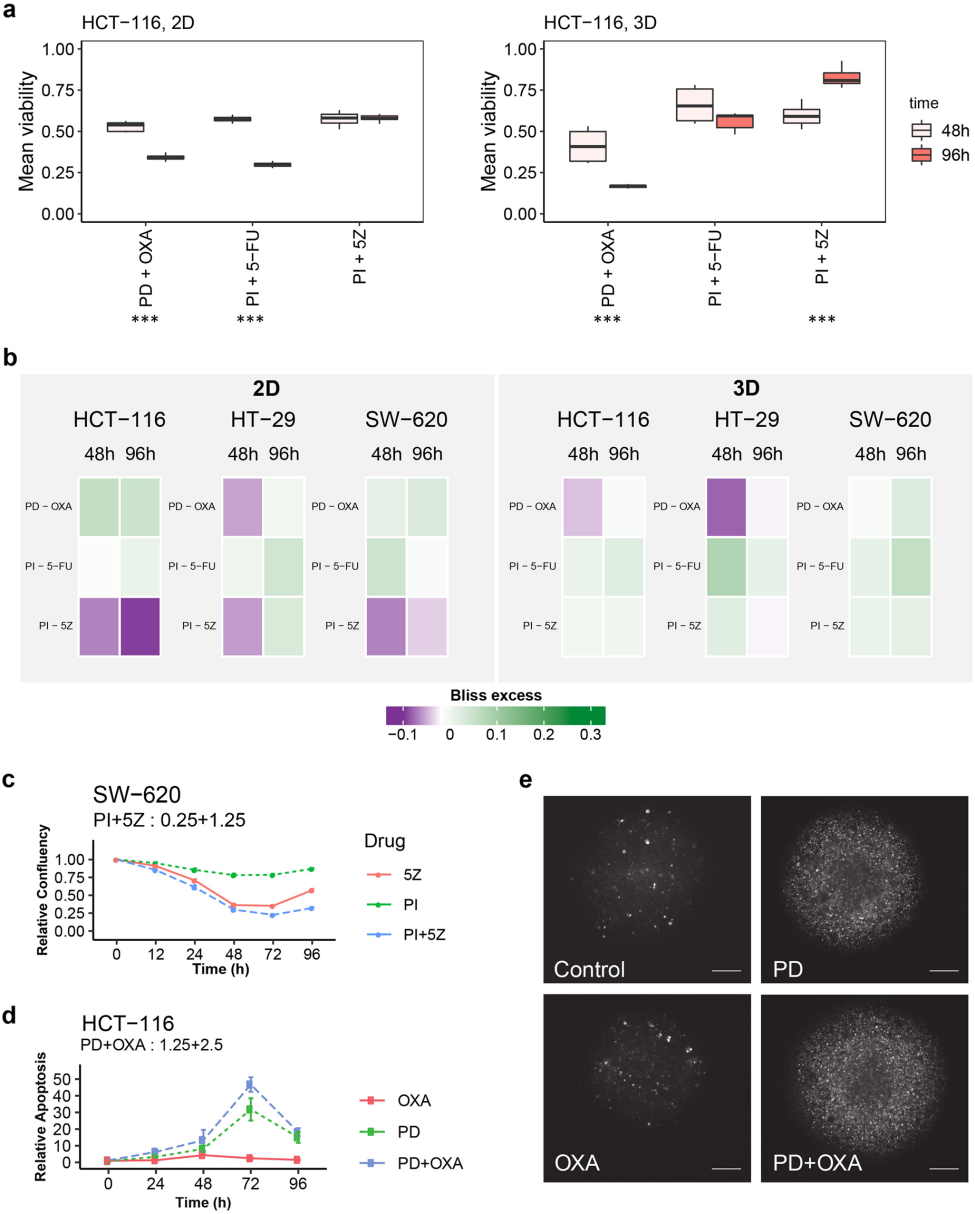
Readout and synergy scoring data upon 48 h and 96 h incubation with drugs. (**a**) Comparison of mean viability at 48 h (combination screen) versus 96 h (96 h screen) for HCT-116 cells cultured in 2D and 3D. Asterisks (*) indicate statistically significant difference in average viability between 48 and 96 h per drug combination, with p ≤ 0.05, p ≤ 0.01 and p ≤ 0.001 for *, ** and ***, respectively. (**b**) Mean Bliss excess at 48 h (combination screen) versus 96 h (96 h screen). (**c**) Relative confluency compared to 0 h and vehicle control in 2D-cultured SW-620 cells exposed to PI and 5Z at the indicated concentrations. (**d**) Relative apoptosis compared to 0 h and vehicle control in 3D-cultured HCT-116 cells treated with the MEK inhibitor PD0325901 (PD), oxaliplatin (OXA), or combination (PD + OXA) at 1.25 (PD) + 2.5 (OXA) µM. (**e**) Representative 72 h-image of apoptotic cells in HCT-116 spheroids treated with vehicle control, 1.25 µM PD, 2.5 µM OXA or combination (PD + OXA). Scale—100 µm. (**c**,**d**) represent the average of three biological replicates with standard deviation.

While in general little to no apoptotic response was observed in any of the cell lines and culture formats upon treatment with the PI3K inhibitor combinations, MEK inhibitor treatment alone induced apoptosis at all concentrations in HCT-116 spheroids (Additional file 9). At high concentrations of oxaliplatin, a further increased apoptotic effect was observed by combination treatment (Fig. 6d,e). This effect was not observed in 2D-cultured HCT-116 cells, in contrast here HT-29 cells showed increased apoptosis under MEK inhibitor treatment, which was also weakly observed in HT-29 spheroids (Additional file 10).

Overall, the correlation between combination drug responses at 48 h and 96 h was strong, with a correlation coefficient (R) ranging from 0.74 to 0.89, and from 0.81 to 0.96 in 2D and 3D cultures, respectively (Fig. S11). When comparing mean viability and combination effect (Bliss excess) at the two time points (48 h and 96 h), we found that although response on average was stronger at 96 h (Fig. 6a, Supplementary Fig. S12), likely due to increased exposure time, combination effects overall decreased across all cell lines (Fig. 6b). This was also reflected in the decreased number of synergistically effective doses in 3 out of 6 conditions (Supplementary Fig. S13). Most striking was the strong effect of MEK inhibition alone, with the highest dose of single PD (1.25 µM) being able to reduce viability to less than 25% across all cell lines, while the combination effect of this inhibitor with oxaliplatin was considerably lower at 96 h compared to 48 h (Fig. 6b). Contrary to the general reduction in mean viability at 96 h compared to 48 h, we observed a significant increase in mean viability of HCT-116 spheroids and SW-620 planar-cultured cells when treated with the PI3K inhibitor in combination with the TAK1 inhibitor (Fig. 6a, Supplementary Fig. S12). This was also reflected by an increase in relative confluency/size in both cell lines, albeit weaker in HCT-116 cells (Fig. 6c).

In summary, these results demonstrate that whereas longer incubation time with drugs not unexpectedly further reduces viability compared to 48 h, the synergistic effect of the tested drug combinations overall tends to be weaker and sometimes even reversed at 96 h. This might be due to already strongly compromised viability at 96 h for each single drug at selected doses, and therefore a much smaller viability range for any synergistic combination response observations. Results from the 96 h exposure screen further support our previous findings of the MEK inhibitor PD0325901 (PD) as a strongly potent inhibitor, which also in single application was able to induce considerable apoptosis in several of the tested conditions (Fig. 6e).

## Discussion

Advances in high-throughput screening and high-content imaging have accelerated testing and discovery of anti-cancer drugs in vitro. However, despite demonstrating efficiency in vitro, only a small fraction of putative treatments has been found to display similar effects in in vivo experiments, and yet fewer in human clinical trials^7,33^. The insufficient ability of in vitro 2D-cultures to recapitulate treatment responses in vivo is believed to be one among many other possible explanations for the slow developmental progress. 3D cell culture models may more closely mimic the architecture of solid tumours and are being anticipated to enable identification of more clinically relevant drug treatments^34^. As a step towards mapping differences and similarities between the two culture formats, we here systematically examined response and combination effects of 7 single-drugs and 21 pairwise combinations in three 2D- and 3D-cultured CRC cell lines (HCT-116, HT-29 and SW-620). While single drug responses have previously been compared in 2D and 3D cultures^9,35,36^, only two other studies have, to our knowledge, compared effects of drug combinations between the two culture formats. The number of combinations tested in these studies has, however, been low (three^37^ and ten^38^ drug combinations, respectively). Furthermore, while Yan et al.^39^ tested 56 drug combinations in 3D cell line cultures, none of these combinations were tested in 2D cultures. To our knowledge, our study represents the largest published comparison of 2D and 3D cultures and their response to cancer-relevant drug combinations.

Altogether, our high-throughput drug screening platform enabled effective identification of single and combination responses in both culture formats. Differences in drug combination responses were observed both between 2D and 3D culture models, readouts and cell lines. This demonstrates the value of including additional readouts and, more so, the use of spheroid-based models for drug combination studies to allow for detection of synergistic effects in different phenotypes and culture formats.

Several studies have reported on altered drug responses in comparisons of spherical versus planar cultures^8,35,37,40–42^. Alterations manifest both as increased and decreased effect of the same drug or drug combination^12^. We too observe culturing mode-related differences in drug sensitivity for some of the tested compounds, with no clear trends pointing towards one of the culture formats as being more sensitive than the other. Consistent with findings by others, we observed reduced sensitivity to the chemotherapeutic agent oxaliplatin in 3D cultures. Riedl et al. previously reported reduced cell cycle progression in several CRC cell lines including HCT-116, HT-29 and SW-620 cells when cultured as spheroids compared to planar cultures^8^, similar to what has also been shown for other cancer types^40^. This accords with our observations of reduced sensitivity of HT-29 spheroids to the cell cycle progression inhibitor palbociclib.

Although the number of studies reporting on differences in single-drug responses between 2D and 3D cultures has been on the rise during the last years, high-throughput drug combination studies are still scarce, with only a few pioneering studies published so far^38,39^. We show that several synergistic drug combinations identified in 2D cultures are not rediscovered in 3D cultures, but also that some synergistic combinations are solely identified in spheroid cultures. In contrast to the observed trend of weaker effect of drug combinations in spheroids, we found that combinations involving the MEK inhibitor PD0325901 exerted a stronger inhibitory effect in 3D cultures. This supports the notion that spheroids show increased dependency on the MEK pathway for their survival^41^. Combinations involving 5Z-7-oxozeanol, which in addition to being a TAK1 inhibitor also has been reported to inhibit MEK1 and ERK2^43^, did in general not show stronger effect in 3D compared to 2D, which could indicate that the inhibitory effect of the TAK1 inhibitor on MEK is considerably weaker than that of PD0325901, in line with other reports^44,45^.

Overall, our results indicate that 2D screening identifies a higher number of positive hits compared to screens of spheroid cultures. This is in contrast to findings by Mathews Griner et al.^38^ who reported a generally higher number of synergistic combinations observed in 3D compared to cells cultured in 2D. Overall, this indicates that there is no general trend in which of the two culture systems appears to be more sensitive to combination treatment. These results thus highlight that when using both culture formats additional interesting combination effects can be observed, that are distinct to one of the culture systems and that would have been missed in screening efforts applying only one of them.

In concordance to findings by Gautam et al.^13^, we notice that readout method matters, underpinned by the fact that we observed several differences in identified drug synergies, synergy strength and combination effect between the different readouts used in this study. The generally high compliance of identified synergies between viability and confluency measurements in 2D cultures can be expected as both assays can be considered as proxies for the number of live cells. The lower compliance of identified synergies between 3D viability and spheroid size might be explained by the generally lower number of observed synergistic effects. Alternatively, differences might be explained by loosening of spheroid structure upon certain treatments, as observed by others^46^ and which might be interpreted as an increase in size, or low effect on cell death by our treatments.

Although synergistic drug combinations called by different readouts (viability vs. confluency/size) overall showed high agreement within culture formats in our screen, the use of imaging-based readouts might still be of high value for assuring technical validity of drug screens, especially when performing drug screens in 3D cultures. While progress in 3D cultivation technologies has simplified the production and handling of spheroids, many cultivation techniques still suffer from limitations associated with generation of uniform spheroids^47^, something that might affect reproducibility of data originating from these models. In our screen, technical as well as biological variability in viability was on average slightly higher in 3D compared to 2D cultures (Supplementary Table S3, Supplementary Fig. S2). As shown by Zanoni et al., both volume and shape of spheroids might affect the response to treatment, in particular when using agents aimed to target proliferating cells^47^. Imaging might allow for pre-selection of optimal spheroids for drug screens by enabling selection of those spheroids meeting specific criteria in terms of e.g. size and morphology^47,48^. By constituting a non-invasive readout method, also real-time monitoring of phenotypic and cellular events is possible^19^, as demonstrated by the continuous measurements of apoptosis (2D, 3D), confluency (2D) and size (3D) in our 96 h screen. Similar to the study by Zanoni et al.^47^, viability data showed relatively high correlation with data from brightfield imaging in our screen (Supplementary Fig. S14), indicating the power of using imaging not only as a backup to the standard viability readout, but also as a possible complement allowing for non-invasive continuous monitoring of drug response.

Today, drug combination screens are commonly performed on large panels of carefully characterised cell lines^6,49^, where combinations considered as clinically relevant often are those classified as synergistic either across the whole panel, or across cell lines in certain mutational-driven clusters. Here, by implementing an approach where drug combinations were mapped according to synergy scores (doses classified as synergistic for Bliss excess < 0) as well as viability response (doses classified as effective for viability ≤ 50%) in 2D and 3D in vitro cultures, we show that the highest scoring drug combinations comprise a sizable number of combinations that are in clinical testing. These results point to the importance of using assessment of cellular phenotype such as viability in addition to synergy score as metrics when evaluating drug combination effects, similarly to what was shown by Meyer et al.^50^. Interestingly, the fourth most synergistically effective drug combination in 3D cultures, 5-FU with the MEK inhibitor, did not demonstrate any synergistically effective doses in 2D cultures, and hence would have been left unidentified if screening in 2D cultures exclusively. The same is true for the two combinations comprising the TAK1 inhibitor with either oxaliplatin or the MEK inhibitor, which were synergistically effective at multiple doses in 3D-cultured HCT-116 and SW-620 cells, but not in 2D-cultured cells. Altogether these results suggest that future screening platforms ideally should encompass monitoring of both conventional ATP-based and additional readouts, as well as more complex culture models, in order to cover as large part of the therapeutic synergy landscape as possible.

## Methods

### Cell lines, drugs and reagents

Human CRC cell lines used in this study were HCT-116 (CVCL_0291), HT-29 (CVCL_0320) and SW-620 (CVCL_0547). The cell lines were directly obtained from NCI. No mycoplasma testing was done in-house. Cells were routinely cultured in 1X RPMI-1640 medium (Thermo Fisher Scientific) supplemented with 10% fetal bovine serum (FBS, Sigma Aldrich), 2 mM l-Glutamine (Sigma Aldrich) and 100 U/mL Penicillin–Streptomycin (Thermo Fisher Scientific). All cells were maintained at 37 °C with 5% CO_2_ and 80% relative humidity and passaged according to in-house protocols (see Supplementary Methods). Cells used in experiments never exceeded passage 21.

Drugs used in screens were olaparib (Selleckchem), oxaliplatin (Selleckchem), palbociclib (Selleckchem), PI-103 (Selleckchem), PD0325901 (Sigma Aldrich), 5-fluorouracil (5-FU, Sigma Aldrich) and 5Z-7-Oxozeaenol (Enzo Life Sciences). Assay reagents used in screens were CellTiter-Glo 2.0 Assay (Promega), CellTiter-Glo 3D Cell Viability Assay (Promega), CellTox Green Cytotoxicity Assay^17^ (Promega) and NucView 488 Caspase-3 Substrate^18^ (Biotium).

### Drug screens

#### Cell seeding procedure

For screening in planar (2D) and spheroid (3D) cultures, cells were plated with 30 µL complete growth medium in 384-well black tissue culture treated plates (Corning) and 384-well black round-bottom ultra-low attachment plates (Corning), respectively. Seeding densities and plating setups are described in Supplementary Methods: Table I. In the 96 h follow-up screen, seeding numbers were reduced for 2D cultures to ensure that controls did not reach full confluency before the endpoint readout. Following seeding, 2D plates were shaken (1,600 rpm, 30 s) to ensure uniform sedimentation of cells. 3D plates were shaken (1,600 rpm, 30 s) and centrifuged (200G, 5 min) to allow aggregation of single cells into spheroids. Before drug addition, cells in 2D and 3D were allowed to adhere/aggregate for 24 h and 72 h, respectively.

#### Drug treatment

Drug compounds and doses used in screens are summarised in Fig. 1a and Supplementary Methods: Table II. For the combination screen, four of the original eight doses screened in single applications were selected (see Supplementary Methods). Drugs in single, combination, vehicle (DMSO, water, DMSO + water 1:1) and positive controls (staurosporine, digitonin) were added in four technical replicates per condition to the wells using a Tecan Freedom EVO robotic system (5 µL/well). For measurement of apoptosis in the 96 h screen, 3 µL of NucView 488 Caspase-3 Substrate (final reagent concentration: 3.43 µM) and 2 µL of drug solution were added to the wells. DMSO concentration never exceeded 0.5%. Cells were incubated (37 °C with 5% CO_2_, 80% relative humidity) with drugs, vehicle, or positive controls for 48 h (single-drug and combination screens) or 96 h (96 h screen).

The single-drug screen was performed with four technical replicates and one biological replicate. The drug combination screen was performed with four technical replicates and two biological replicates per condition. The drug combination PD + 5Z at doses 0.05 µM + 0.01 µM was excluded from biological replicate 1 as no drug was added to the wells due to a robotic error. The 96 h screen was performed with 2–4 technical replicates and three biological replicates.

#### Readouts

All readouts are listed in Supplementary Methods: Table III. Shortly, for 2D-cultured cells confluency was assessed based on brightfield imaging. Apoptosis was assessed using NucView 488 Caspase-3 Substrate and fluorescence imaging (excitation: 456 nm, emission: 541 nm). Cell death (membrane integrity) was monitored using CellTox Green Cytotoxicity Assay by reading fluorescence at 535 nm. Cell viability was measured by reading luminescence after 10 min incubation with CellTiter-Glo 2.0 reagent (20 µL/well, mixed 1:1 with PBS prior to addition). A SpectraMax i3x reader equipped with a MiniMax 300 Imaging Cytometer (Molecular Devices) was used for all 2D readouts and image analysis.

Spheroid viability was measured by reading luminescence (Tecan infinite M200 Pro) after 60 min incubation with CellTiter-Glo 3D reagent (20 µL/well). Preceding addition of the CellTiter-Glo 3D reagent, images (× 4 magnification) were captured using an EVOS 1 imaging system (single-drug screen) or an ImageXpress Micro Confocal High-Content Imaging System (Molecular Devices). Apoptosis in spheroids was monitored using NucView 488 Caspase-3 Substrate and confocal fluorescence imaging. Fluorescent Z stack images (five planes per stack and 50 µm separation between planes at 0-72 h; ten planes per stack and 10 µm separation between planes at 96 h) were captured continuously. At each time point, spheroid size was estimated using brightfield imaging of mid-planes.

### Data processing and statistical analysis

Confluency and apoptosis (2D) were estimated by re-analysing brightfield and fluorescence images using the SoftMax Pro 6 software. For each well, percentage of covered area (confluency) and number of fluorescent objects (apoptosis) were estimated. Spheroid size was quantified by high-throughput size measurement using SpheroidSizer^51^ in Matlab (The MathWorks Inc., Natick, Massachusetts) version 2017a (single-drug screen) or 2015a (combination and 96 h screen). Apoptosis in spheroids was quantified by estimating the number of fluorescent cells in imaged sections using the MetaXpress software. All treatment effects are normalised to the internal vehicle control per plate and reported as average ± standard deviation. Pearson’s correlation coefficient (R) has been used to quantify the association between variables.

R versions 3.5.1 and 3.5.3 were used for data processing and graphics, respectively. Packages are summarised in Supplementary Methods: Table IV. For statistical analyses, a two-tailed Student’s *t* test (with p < 0.05 being considered significant) has been used when comparing two groups.

### Synergy scoring

The Bliss independence reference model^22^ was used to estimate synergy. The Bliss expectation (E_AB, Bliss_) is calculated for drugs A and B from effect (E) as E_AB, Bliss_ = E_A_ + E_B_ − E_A_E_B_, and synergy is called if the observed effect of the combination is larger than the expectation. Synergy scores were calculated per biological replicate, followed by calculation of mean synergy scores across biological replicates as presented in^52^. We report both average and standard deviation of Bliss excess values per dose and biological replicate, as well as across the matrix.

### Screen reproducibility

Inter- and intra-experiment reproducibility of response was assessed by comparing data points (doses) common for the different setups (Pearson correlation). The correlation coefficients for single-drug responses (viability data) in the single-drug screen and the combination screen were 0.78 and 0.77, for 2D and 3D cultures, respectively (Supplementary Fig. S1). The intra-experiment reproducibility for the combination screen was assessed based on the two biological replicates. Correlation coefficients were 0.97, 0.92, 0.93 and 0.95 for 2D viability, 2D confluency, 3D viability and spheroid size, respectively (Supplementary Fig. S2). The intra-experiment reproducibility for the 96 h screen was assessed based on three biological replicates. Correlation coefficients (viability data) ranged from 0.96 to 0.99 (Supplementary Fig. S3). Technical variability was assessed by computing the Coefficient of Variation (CV) per condition (treatment), biological replicate and readout. An overall CV was calculated by averaging the CV values per biological replicate and readout (Supplementary Table S3).

## Supplementary information

Supplementary Information.

## Data Availability

All data supporting the conclusions of this article are available in the Figshare repository (https://figshare.com/s/b2b0726049f10a763e39).
