# Author Correction: High-throughput screening reveals higher synergistic effect of MEK inhibitor combinations in colon cancer spheroids

**DOI:** 10.1038/s41598-020-77501-4

**Published:** 2020-11-25

**Authors:** Evelina Folkesson, Barbara Niederdorfer, Vu To Nakstad, Liv Thommesen, Geir Klinkenberg, Astrid Lægreid, Åsmund Flobak

**Affiliations:** 1 Department of Clinical and Molecular Medicine, Norwegian University of Science and Technology, Trondheim, Norway; 2 The Cancer Clinic, St Olav’s University Hospital, Trondheim, Norway; 3 Department of Biotechnology, SINTEF Materials and Chemistry, Trondheim, Norway; 4 Department of Biomedical Laboratory Science, Norwegian University of Science and Technology, Trondheim, Norway

Correction to: *Scientific Reports* 10.1038/s41598-020-68441-0, published online 14 July 2020

The original version of this Article contained errors in the Reference list. References 46–50 were incorrectly listed as References 44–48 and References 51–52 were incorrectly listed as References 49–50 respectively.

In addition, References 44 and 45 were omitted. References 44 and 45 are listed below:

Bain, J. *et al.* The selectivity of protein kinase inhibitors: A further update. *Biochem. J.* **408**, 297–315 (2007).

Ninomiya-Tsuji, J. *et al.* A resorcylic acid lactone, 5Z-7-oxozeaenol, prevents inflammation by inhibiting the catalytic activity of TAK1 MAPK kinase kinase. *J. Biol. Chem.* **278**, 18485–18490 (2003).

Furthermore, the Article contained the following typographical errors in the Reference citations.

In the Discussion section,

“Today, drug combination screens are commonly performed on large panels of carefully characterised cell lines^4,33^, where combinations considered as clinically relevant often are those classified as synergistic either across the whole panel, or across cell lines in certain mutational-driven clusters.”

now reads:

“Today, drug combination screens are commonly performed on large panels of carefully characterised cell lines^6,49^, where combinations considered as clinically relevant often are those classified as synergistic either across the whole panel, or across cell lines in certain mutational-driven clusters.”

“These results point to the importance of using assessment of cellular phenotype such as viability in addition to synergy score as metrics when evaluating drug combination effects, similarly to what was shown by Meyer et al.^34^.”

now reads:

“These results point to the importance of using assessment of cellular phenotype such as viability in addition to synergy score as metrics when evaluating drug combination effects, similarly to what was shown by Meyer et al.^50^.”

In the Methods section,

“Assay reagents used in screens were CellTiter-Glo 2.0 Assay (Promega), CellTiter-Glo 3D Cell Viability Assay (Promega), CellTox Green Cytotoxicity Assay^15^ (Promega) and NucView 488 Caspase-3 Substrate^18^ (Biotium).”

now reads:

“Assay reagents used in screens were CellTiter-Glo 2.0 Assay (Promega), CellTiter-Glo 3D Cell Viability Assay (Promega), CellTox Green Cytotoxicity Assay^17^ (Promega) and NucView 488 Caspase-3 Substrate^18^ (Biotium).”

Lastly, in the Supplementary Information file of this Article, Evelina Folkesson and Astrid Lægreid were incorrectly affiliated with ‘The Cancer Clinic, St Olav’s University Hospital, Trondheim, Norway’. The correct affiliation is given below:

‘Department of Clinical and Molecular Medicine, Norwegian University of Science and Technology, Trondheim, Norway.’

These errors have now been corrected in the PDF and HTML versions of the Article, and in the accompanying Supplementary Information file.

