# Supplementary material for: High-throughput screening reveals higher synergistic effect of MEK inhibitor combinations in colon cancer spheroids
Source: Sci Rep. 2020 Jul 14;10:11574. doi: 10.1038/s41598-020-68441-0 (PMC7360566; doi:10.1038/s41598-020-68441-0)
Supplement: Supplementary file 1 — Supplementary Information. [file 41598_2020_68441_MOESM1_ESM.docx]

Supplementary Material

High-throughput screening reveals higher synergistic effect of MEK inhibitor combinations in colon cancer spheroids

Evelina Folkesson^1^†, Barbara Niederdorfer^1^†, Vu To Nakstad^3^, Liv Thommesen^4^, Geir Klinkenberg^3^, Astrid Lægreid^1^, Åsmund Flobak^1,2*^

^1^ Department of Clinical and Molecular Medicine, Norwegian University of Science and Technology, Trondheim, Norway

^2^ The Cancer Clinic, St Olav’s University Hospital, Trondheim, Norway

^3^ SINTEF Materials and Chemistry, Department of Biotechnology, Trondheim, Norway

^4^ Department of Biomedical Laboratory Science, Norwegian University of Science and Technology, Trondheim, Norway

†These authors contributed equally to this work

*asmund.flobak@ntnu.no

Additional file 1

# **Supplementary Methods**

## Cell passaging

Long-term stored in liquid nitrogen, HCT-116, HT-29 and SW-620 cells were thawed and passaged for at least two weeks (split twice a week) before entering experiments. The standard splitting procedure included i) aspiration of old medium, ii) PBS wash (1-2 á 12 mL), iii) trypsinization (Thermo Fisher Scientific), 5 minutes at 37°C, iv) resuspension in complete growth medium (RPMI Medium 1640 (1X) supplemented with 10% fetal bovine serum (FBS), 2 mM L-Glutamine and 100 U/mL Penicillin-Streptomycin). A fraction of cells (1:10, 1:8 and 1:6 for HCT-116, HT-29 and SW-620 cells, respectively) was transferred to a new T75 flask. Final volume in T75 flask: 15 mL. Cells were passaged twice per week.

## Selection of doses

Doses for the combination screen were guided by the IC20 for each single-drug observed with CellTiter-Glo in 2D and 3D cultures. IC20 calculations were performed in R (version 3.3.3) using the drc package version 3.0.1 and a five-parameter log-logistic dose-response model^1^. In cases where the lowest drug concentration used in the single-drug screen reduced viability with more than 20%, the four lowest screened doses from the single-drug screen were selected for the combination screen. In cases where the highest drug concentration reduced viability with less than 20%, the four highest screened doses from the single-drug screen were selected. In remaining cases, we selected the 4-points dose range that covered as many of the calculated IC20 values as possible for that specific drug. Note that for each drug, the same doses were selected for all cell lines in 2D and 3D.

Table I. Seeding numbers and assay reagent concentrations (at plating). Setup 1 = cells in medium, Setup 2 = cells in medium + CellTox Green, Setup 3 = cells in medium + NucView 488 Caspase-3 Substrate. * Cells (2D, 3D) seeded in medium (Setup 1) - NucView 488 Caspase-3 Substrate added at drug addition.

| **Cell line** | **Seeding density (cells/well); assay reagent concentration** | | | | | | | |
| --- | --- | --- | --- | --- | --- | --- | --- | --- |
|  | **Single-drug screen** | | | | **Combination screen** | | **96 hours screen*** | |
|  | **2D** | | | **3D** | **2D** | **3D** | **2D** | **3D** |
|  | **Setup 1** | **Setup 2** | **Setup 3** | **Setup 1** | **Setup 1** | **Setup 1** | **Setup 1** | **Setup 1** |
| HCT-116 | 1200;  - | 1200; 1:1000 | 1200;  3.43 µM | 1200;  - | 1200;  - | 1200;  - | 300;  - | 1200;  - |
| HT-29 | 3750;  - | 3750;  1:1000 | 3750;  3.43 µM | 4800;  - | 3750;  - | 4800;  - | 900;  - | 4800;  - |
| SW-620 | 3000;  - | 3000;  1:1000 | 3000;  3.43 µM | 600;  - | 3000;  - | 600;  - | 900;  - | 600;  - |

Table II. Compounds and doses used in the single-drug (SDS), combination (CS) and 96 hours screen (96h).
* Primary target according to manufacturer.

| **Compound** | **Abbreviation** | **Primary target*** | **Solvent** | **Doses (µM)** | | | | **PubChem CID** |
| --- | --- | --- | --- | --- | --- | --- | --- | --- |
|  |  |  |  | **SDS** | **CS** | | **96h** |  |
| Olaparib | OLA | PARP1,  PARP2 | DMSO | 0.01, 0.05, 0.25, 1.25, 2.5, 5.0, 10,  20 | 2.5,  5,  10,  20 | Not included | | 23725625 |
| Palbociclib | PAL | CDK4,  CDK6 | Water |  | 0.25, 1.25, 2.5,  5 | Not included | | 11478676 |
| PD0325901 | PD | MEK1,  MEK2 | DMSO |  | 0.01, 0.05, 0.25, 1.25 | 0.01, 0.05, 0.25, 1.25 | | 9826528 |
| PI-103 | PI | PI3K  (p110α/β/γ/δ) | DMSO |  | 0.01, 0.05, 0.25, 1.25 | 0.01, 0.05, 0.25, 1.25 | | 9884685 |
| 5Z-7-  Oxozeanol | 5Z | TAK1 | DMSO |  | 0.01, 0.05, 0.25, 1.25 | 0.01, 0.05, 0.25, 1.25 | | 9863776 |
| 5-Fluorouracil | 5-FU | Thymidylate  synthase | DMSO |  | 2.5,  5,  10,  20 | 2.5,  5,  10,  20 | | 3385 |
| Oxaliplatin | OXA | DNA synthesis | Water |  | 0.05, 0.25, 1.25, 2.5 | 0.05, 0.25, 1.25,  2.5 | | 4609 |

Table III. Readouts per culture format in single-drug (SDS), combination (CS) and 96 hours screen (96h). Assays, as well as readout time points, are indicated. Confluency and apoptosis were assessed by imaging two views per well.

| **Culture format** | **Readout** | **Assay reagent** | **Detection method** | **Time point (h)** | | | | |
| --- | --- | --- | --- | --- | --- | --- | --- | --- |
|  |  |  |  | **SDS** | **CS** | | **96h** | |
| 2D | Viability | CellTiter-Glo 2.0 | Luminescence | 48 | | 48 | | 96 |
|  | Confluency | - | Brightfield imaging | 0, 6,  12, 18,  24, 30,  36, 48 | | 48 | | 0, 12,  24, 48,  72, 96 |
|  | Cell death | CellTox Green | Fluorescence | 0, 6,  12, 18,  24, 30,  36, 48 | | - | | - |
|  | Apoptosis | NucView 488 | Fluorescence imaging | 0, 6,  12, 18,  24, 30,  36, 48 | | - | | 0, 12,  24, 48,  72, 96 |
| 3D | Viability | CellTiter-Glo 3D | Luminescence | 48 | | 48 | | 96 |
|  | Size | - | Brightfield imaging | 0, 48 | | 48 | | 0, 24,  48, 72,  96 |
|  | Apoptosis | NucView 488 | Fluorescence imaging | - | | - | | 0, 24,  48, 72,  96 |

Table IV. R packages used for data processing and graphics.

| **Execution** | **Package** | **Version** | **Comment** |
| --- | --- | --- | --- |
| Data processing | tidyr | 0.8.3 | R version 3.5.1 |
|  | dplyr | 0.8.1 | R version 3.5.1 |
|  | lattice | 0.20.38 | R version 3.5.1 |
|  | PharmacoGx | 1.12.0 | R version 3.5.2 |
| Graphics | ggplot2 | 3.2.0 | R version 3.5.3 |
|  | ComplexHeatmap^2^ | 1.20.0  2.3.2 | R version 3.5.3 |
|  | ggpubr | 0.2 | R version 3.5.3 |
|  | gridExtra | 2.3 | R version 3.5.3 |
|  | ggrepel | 0.8.1 | R version 3.5.3 |
|  | grid | 3.5.3 | R version 3.5.3 |
|  | UpsetR^3^ | 1.4.0 | R version 3.5.3 |

# **References**

1. Gottschalk, P. G. & Dunn, J. R. The five-parameter logistic: A characterization and comparison with the four-parameter logistic. *Anal. Biochem.* **343**, 54–65 (2005).

2. Gu, Z., Eils, R. & Schlesner, M. Complex heatmaps reveal patterns and correlations in multidimensional genomic data. *Bioinformatics* **32**, 2847–2849 (2016).

3. Lex, A., Gehlenborg, N., Strobelt, H., Vuillemot, R. & Pfister, H. UpSet: Visualization of Intersecting Sets. *IEEE Trans. Vis. Comput. Graph.* **20**, 1983–1992 (2014).

Additional file 2

# **Supplementary Tables**

Table S1 - IC20 (µM) values estimated from viability data (single-drug screen). Non-computable IC20 values are indicated by NaN in the table. Estimated IC20 values outside screening range (0.01-20 µM) are indicate by < D.r. and > D.r..

|  | **IC20 (µM)** | | | | | |
| --- | --- | --- | --- | --- | --- | --- |
| **Drug** | **HCT-116** | | **HT-29** | | **SW-620** | |
|  | **2D** | **3D** | **2D** | **3D** | **2D** | **3D** |
| **OLA** | NaN | NaN | NaN | < D.r. | NaN | NaN |
| **PAL** | 2.33 ± 0.85 | 0.47 ± 2.13 | 5.26 ± 0.17 | NaN | 2.70 ± 3.78 | 0.013 ± 0.47 |
| **PD** | 0.02 ± 0.33 | < D.r. | < D.r. | 0.01 ± 0.02 | < D.r. | < D.r. |
| **PI** | 0.41 ± 0.11 | 0.93 ± 0.12 | > D.r | NaN | 0.38 ± 0.05 | 0.10 ± NA |
| **5Z** | 0.29 ± 0.06 | 0.07 ± 0.04 | 0.06 ± NA | 0.01 ± NA | 0.45 ± 0.09 | 0.06 ± NA |
| **5-FU** | 3.49 ± 0.52 | 3.28 ± 0.62 | 11.58 ± 2.65 | NaN | 13.60 ±7.34 | NaN |
| **OXA** | 0.47 ± 0.07 | 1.01 ± 0.16 | 2.90 ± NA | < D.r. | 0.06 ± 0.09 | 0.26 ± 0.05 |

Table S2 – Synergistic drug combinations (x) per readout and cell line. Average Bliss score per 5x5 matrix was calculated for all drug combinations per readout and cell line. Imaging corresponds to assessment of confluency and size in 2D and 3D cultures, respectively.

| **Synergistic drug combinations** | **HCT-116** | | | | **HT-29** | | | | **SW-620** | | | |
| --- | --- | --- | --- | --- | --- | --- | --- | --- | --- | --- | --- | --- |
|  | **Viability** | | **Imaging** | | **Viability** | | **Imaging** | | **Viability** | | **Imaging** | |
|  | **2D** | **3D** | **2D** | **3D** | **2D** | **3D** | **2D** | **3D** | **2D** | **3D** | **2D** | **3D** |
| 5Z + PAL | X |  | X | X | X |  | X |  | X |  | X |  |
| 5Z + PI | X |  | X |  | X |  | X | X | X |  | X |  |
| PD + OLA | X | X | X |  |  |  | X |  |  | X |  | X |
| PD + PI | X | X | X |  | X |  | X | X |  |  |  |  |
| 5-FU + PAL | X | X | X | X |  |  |  |  |  |  |  |  |
| PD + OXA |  | X |  |  | X | X |  | X |  |  |  |  |
| PD + PAL | X |  | X |  | X |  | X |  |  |  |  |  |
| OLA + 5Z | X |  |  |  |  | X | X |  |  |  |  |  |
| OXA + PAL | X |  | X |  | X |  |  |  |  |  |  |  |
| PAL + OLA | X | X | X |  |  |  |  |  |  |  |  |  |
| PI + OLA | X |  | X |  |  |  |  |  |  |  |  | X |
| 5Z + OXA |  |  |  |  | X |  |  | X |  |  |  |  |
| OLA + 5-FU |  | X |  |  |  |  |  |  |  | X |  |  |
| OXA + 5-FU |  |  |  |  | X |  | X |  |  |  |  |  |
| 5-FU + PD |  |  |  |  |  | X |  |  |  |  |  |  |
| 5-FU + PI |  |  |  |  |  |  | X |  |  |  |  |  |
| 5Z + 5-FU |  |  |  |  |  |  | X |  |  |  |  |  |

Table S3 – Average Coefficient of Variation (%) per biological replicate and readout. Average CV values were calculated by averaging those per condition (treatment) within each biological replicate and readout.

| **Readout** | **Average CV (%)** | |
| --- | --- | --- |
|  | **Rep 1** | **Rep 2** |
| Viability 2D | 6.01 | 6.90 |
| Confluency 2D | 3.03 | 4.95 |
| Viability 3D | 9.59 | 9.55 |
| Size 3D | 4.19 | 3.88 |

# **Supplementary Figures**

[Figure S1 - Inter-experiment reproducibility for single-drug and combination screens. 9](#_Toc35677449)

[Figure S2 - Intra-experiment reproducibility for the combination screen 10](#_Toc35677450)

[Figure S3 - Intra-experiment reproducibility for the 96 hours screen 11](#_Toc35677451)

[Figure S4 - Single-drug dose-response viability data (endpoint, 48h) 12](#_Toc35677452)

[Figure S5 - Single-drug dose-response confluency data 13](#_Toc35677453)

[Figure S6 - Single-drug dose-response cell death data (continuous) 14](#_Toc35677454)

[Figure S7 - Single-drug dose-response spheroid size data (endpoint, 48h) 15](#_Toc35677455)

[Figure S8 - Average viability in the combination screen (endpoint, 48h) 16](#_Toc35677456)

[Figure S9 - Venn diagrams showing the number of synergistic drug combinations identified by one or more readouts 17](#_Toc35677457)

[Figure S10 - Number of synergistic doses per combination, cell line and culture format, which reduce confluency or spheroid size to < 0.7 18](#_Toc35677458)

[Figure S11 - Combination and 96 hours screen viability and Bliss excess correlation 19](#_Toc35677459)

[Figure S12 - Average viability per combination in the combination and 96 hours screens 20](#_Toc35677460)

[Figure S13 - Number of synergistically effective doses per combination, cell line and culture format at 48h (high-throughput screen) and 96h (96 hours screen). 20](#_Toc35677461)

[Figure S14 - Correlation between relative readout response in the combination screen in 2D, and 3D-cultured cells. 21](#_Toc35677462)


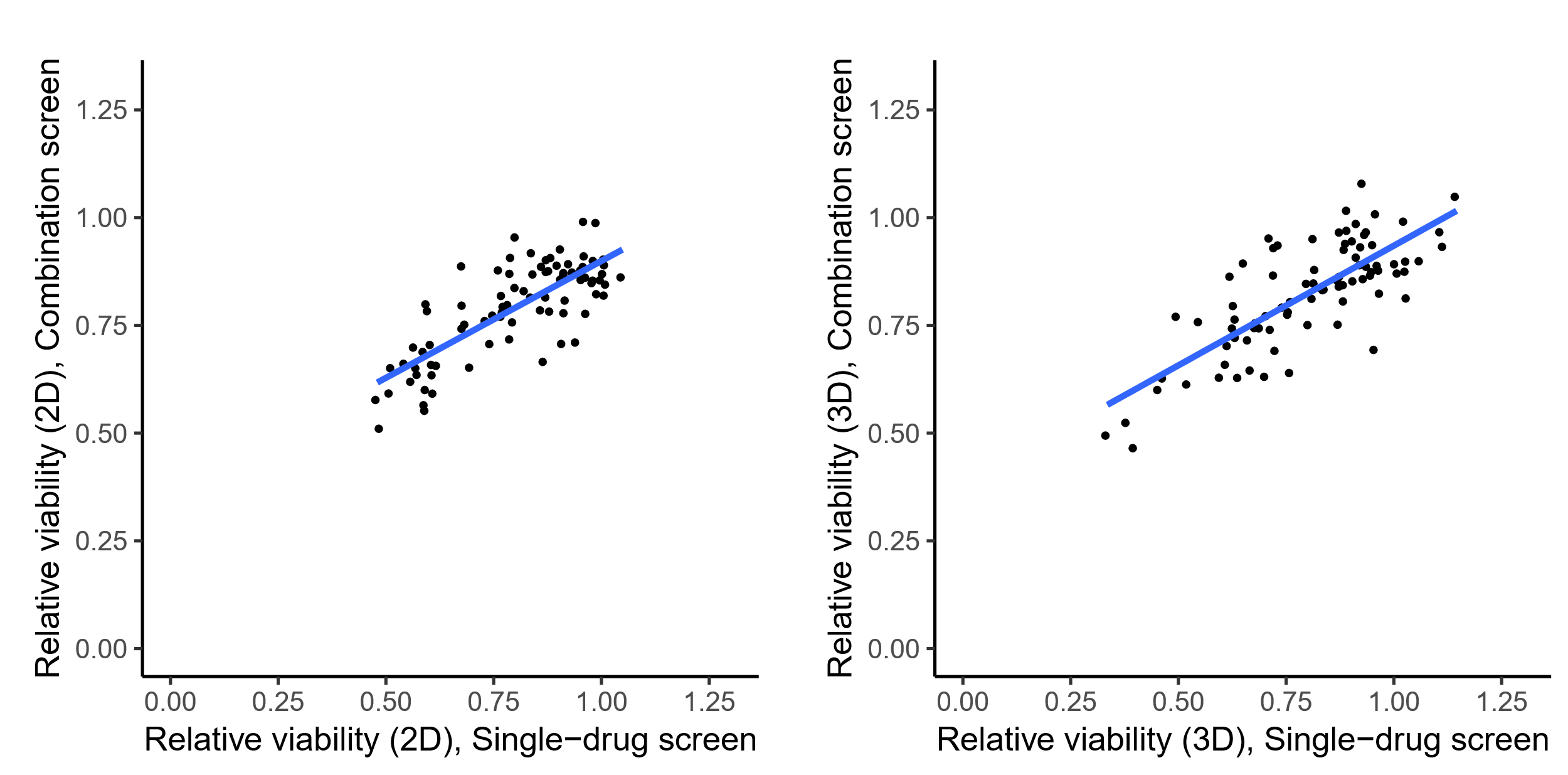


Figure S1 - Inter-experiment reproducibility for single-drug and combination screens. (a) Correlation plots showing Pearson’s correlation between data points (viability) common for the single-drug dose-response screen and the combination screen in 2D and (b) 3D.


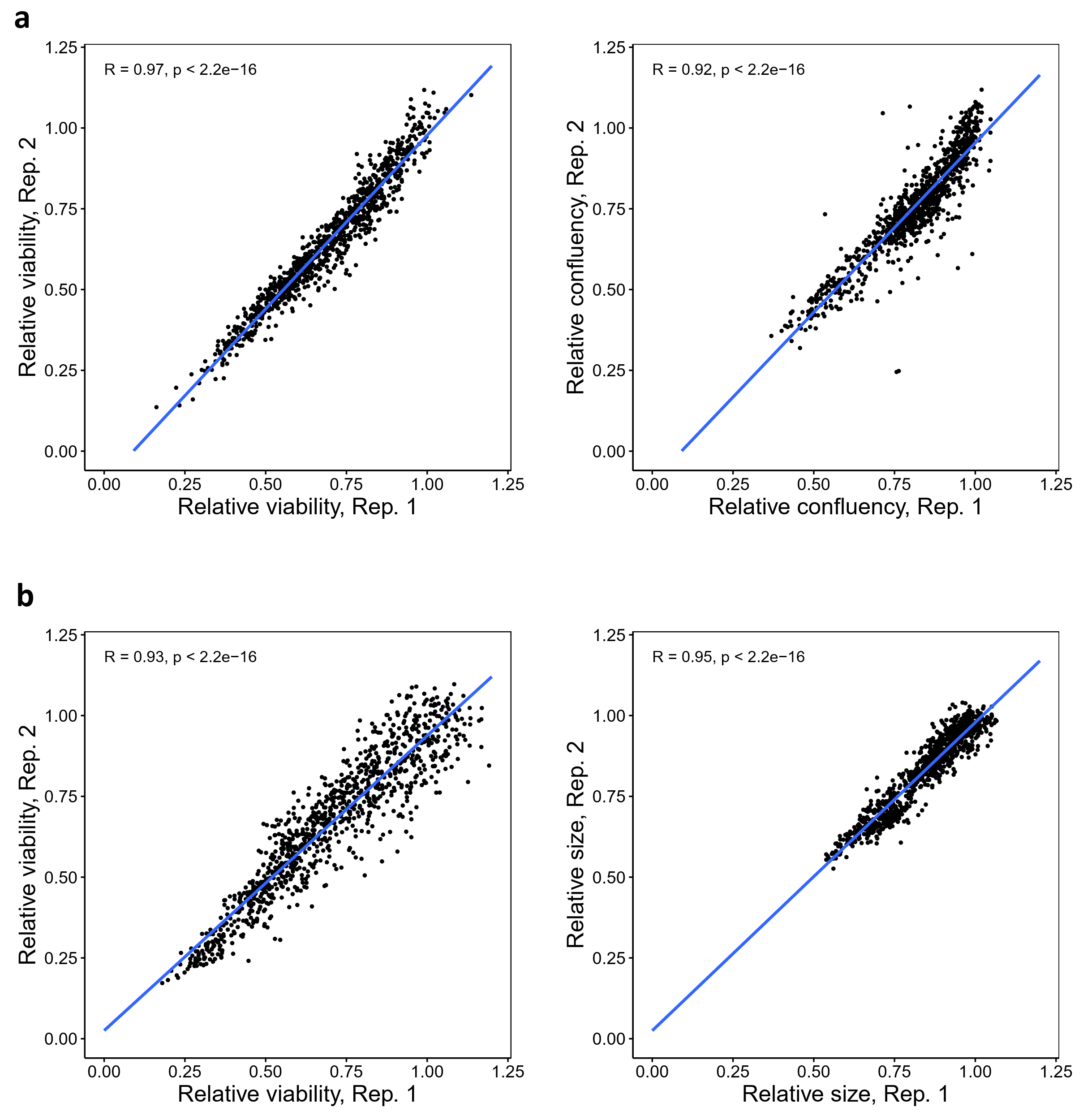


Figure S2 - Intra-experiment reproducibility for the combination screen. (a) Correlation plots showing Pearson’s correlation between replicates per readout in 2D and (b) 3D.


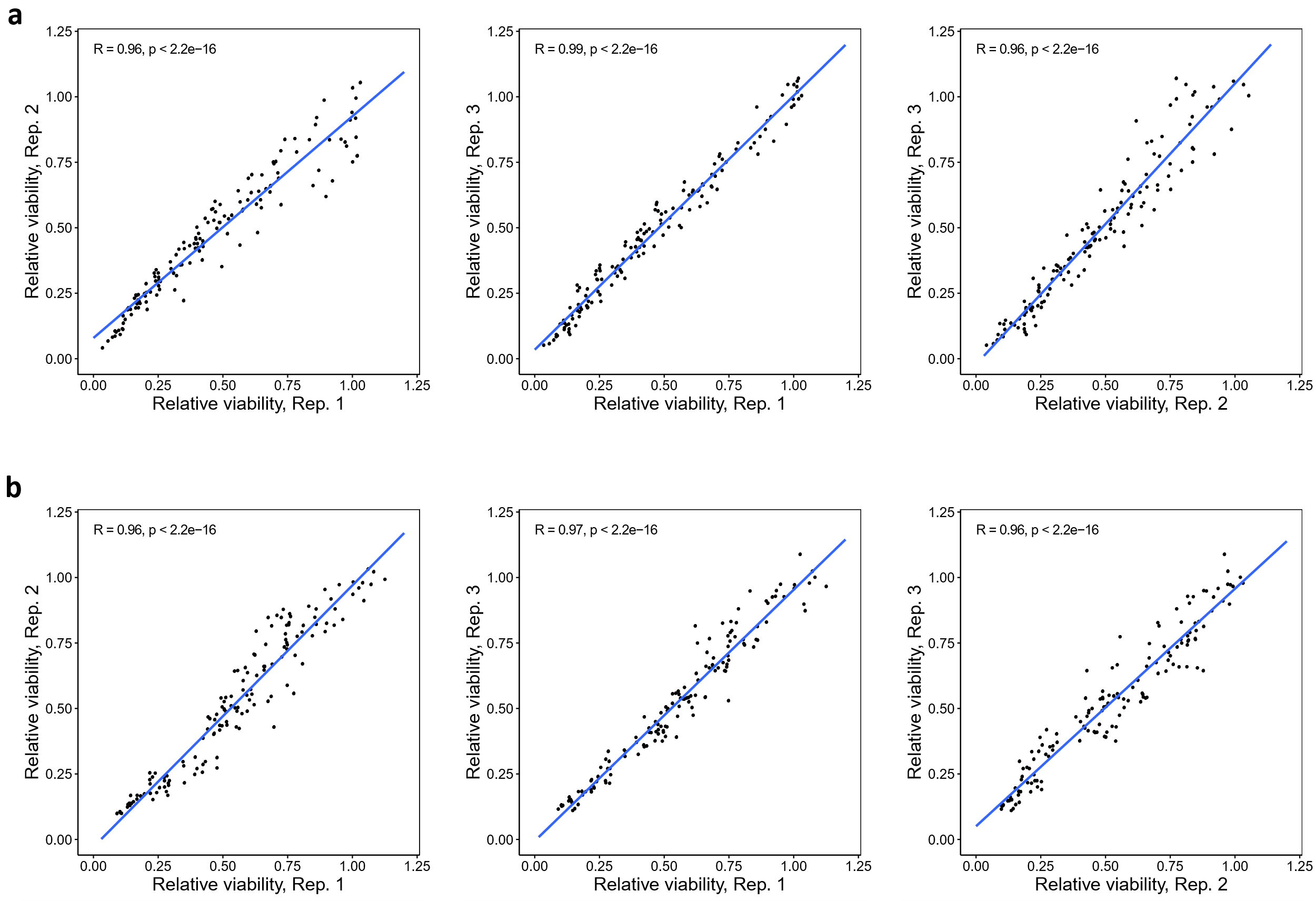


Figure S3 - Intra-experiment reproducibility for the 96 hours screen. (a) Correlation plots showing Pearson’s correlation between replicates (viability) in 2D and (b) 3D.


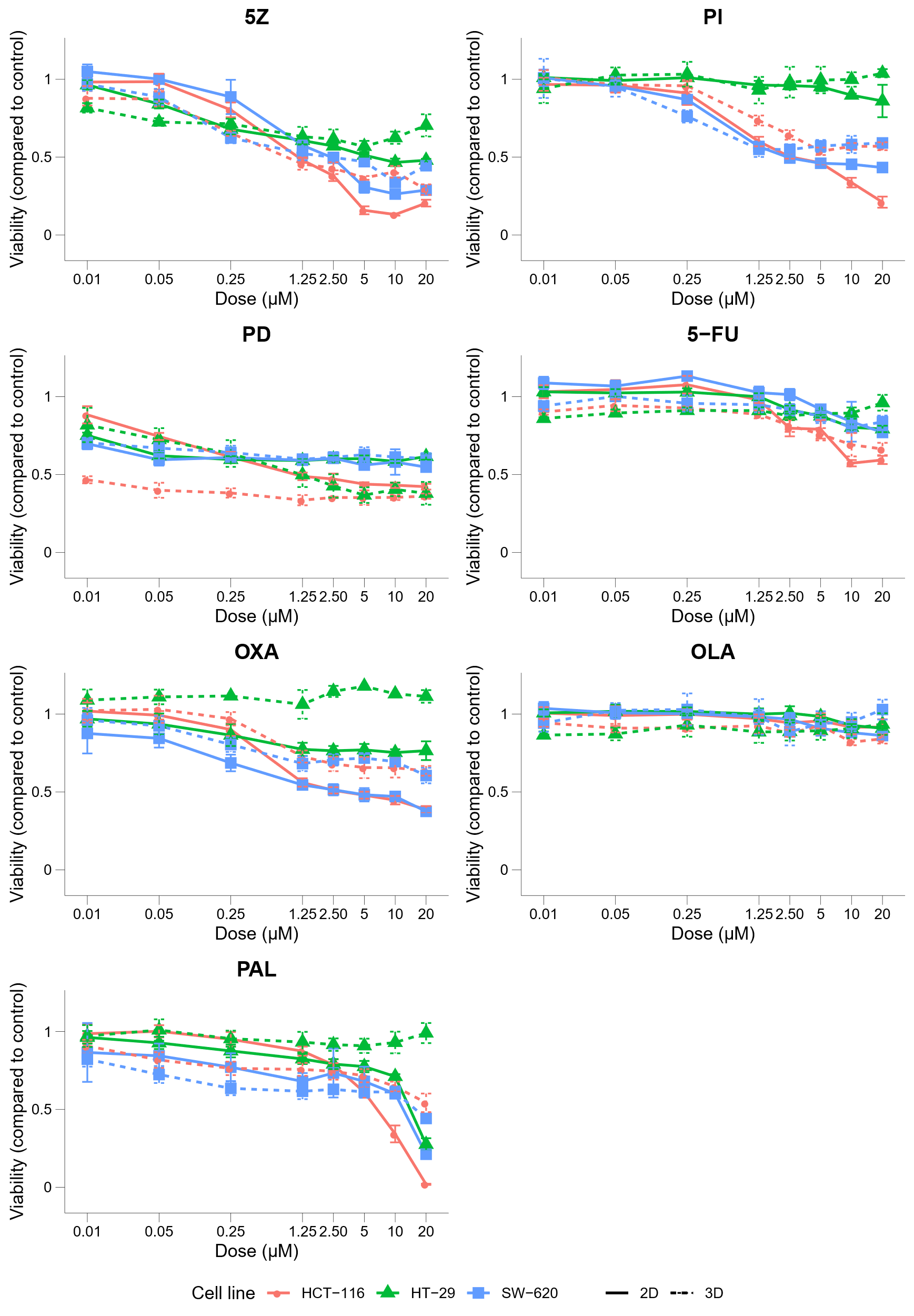


Figure S4 - Single-drug dose-response viability data (endpoint, 48h). Relative viability of HCT-116, HT-29 and SW-620 cells upon exposure to seven single compounds as measured by CellTiter-Glo.


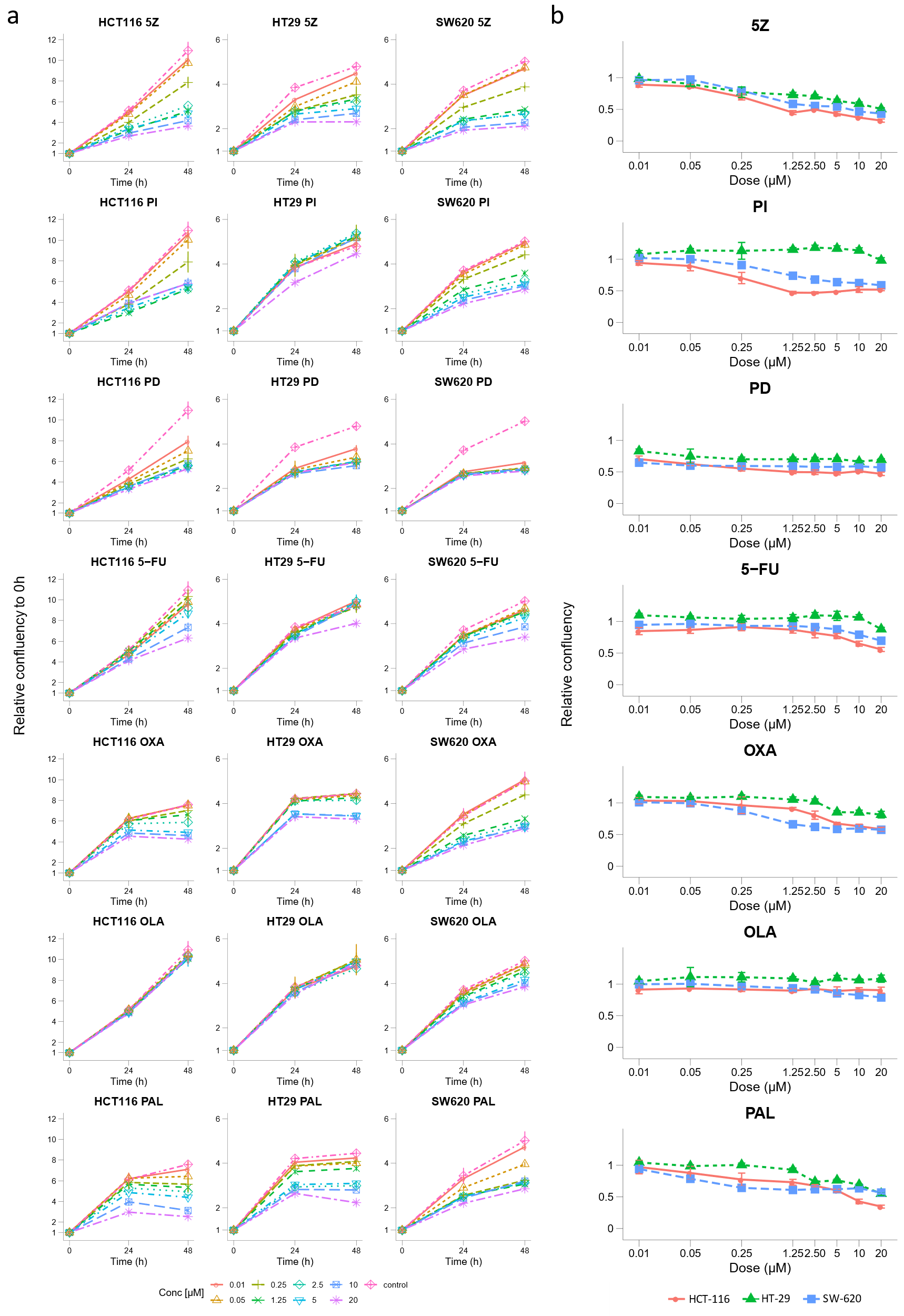


Figure S5 - Single-drug dose-response confluency data. Cellular confluency of HCT-116, HT-29 and SW-620 cells upon exposure to seven single-compounds as measured by brightfield imaging. (a) Continuous confluency compared to 0h. Control shows cell growth of internal plate control. (b) Relative confluency at endpoint (48h) compared to vehicle control and 0h.


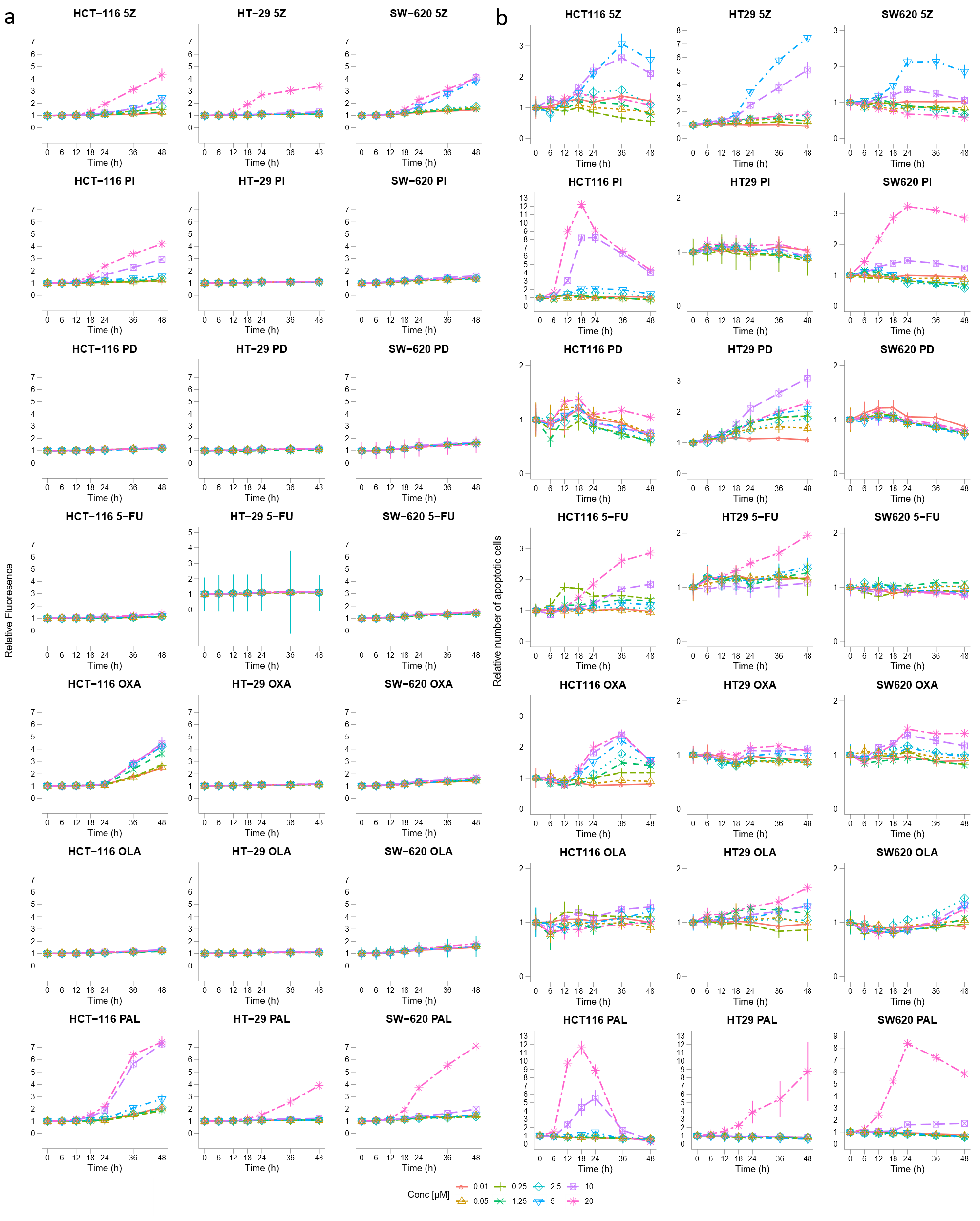


Figure S6 - Single-drug dose-response cell death data (continuous). (a) Relative cell death of HCT-116, HT-29 and SW-620 cells upon exposure to seven single-compounds at doses 0.01 – 20 µM, as measured by CellTox-Green (cell membrane integrity) and (b) NucView (apoptosis).


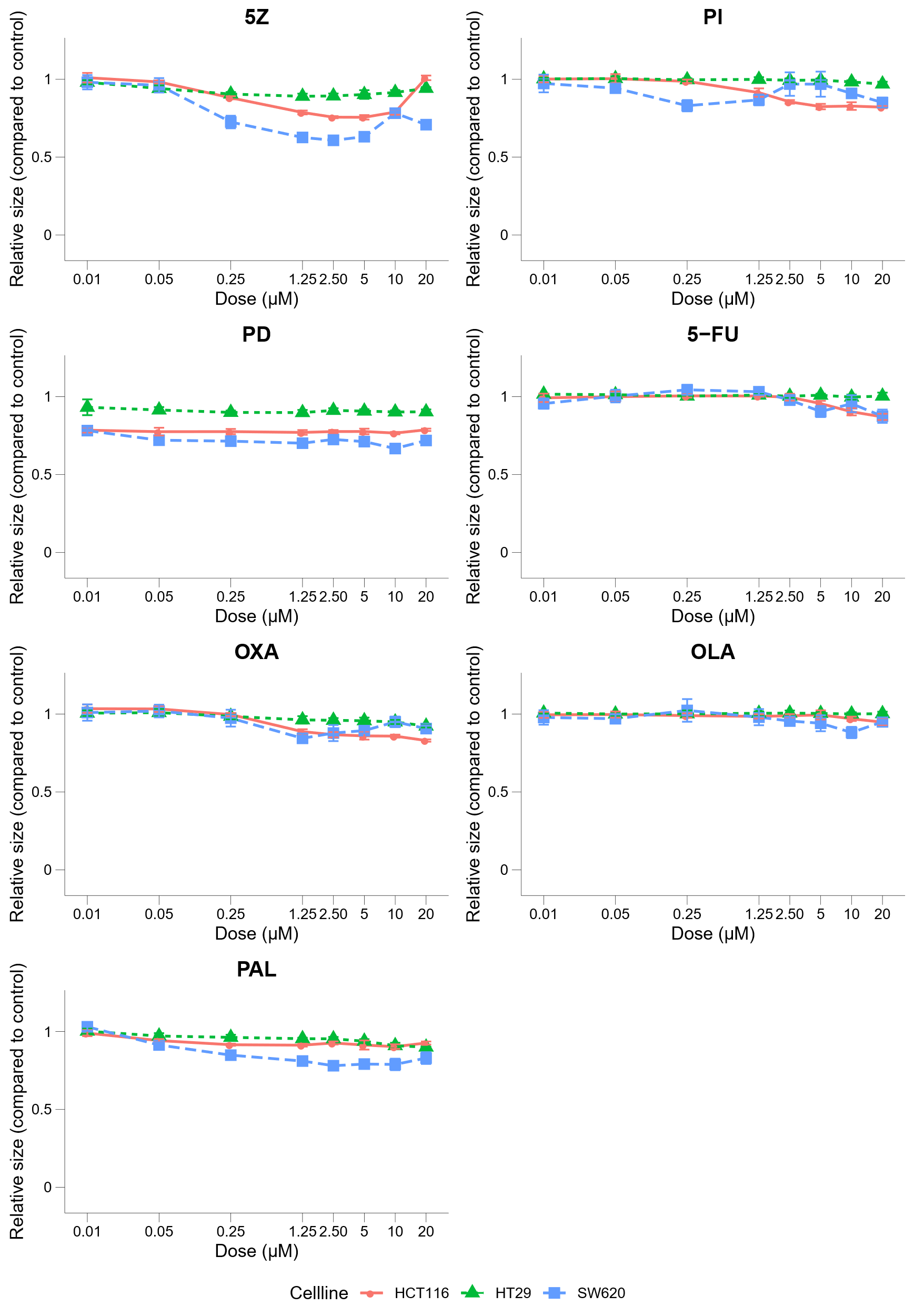


Figure S7 - Single-drug dose-response spheroid size data (endpoint, 48h). Relative spheroid size of HCT-116,
HT-29 and SW-620 spheroids upon exposure to seven single-compounds as measured by brightfield imaging.


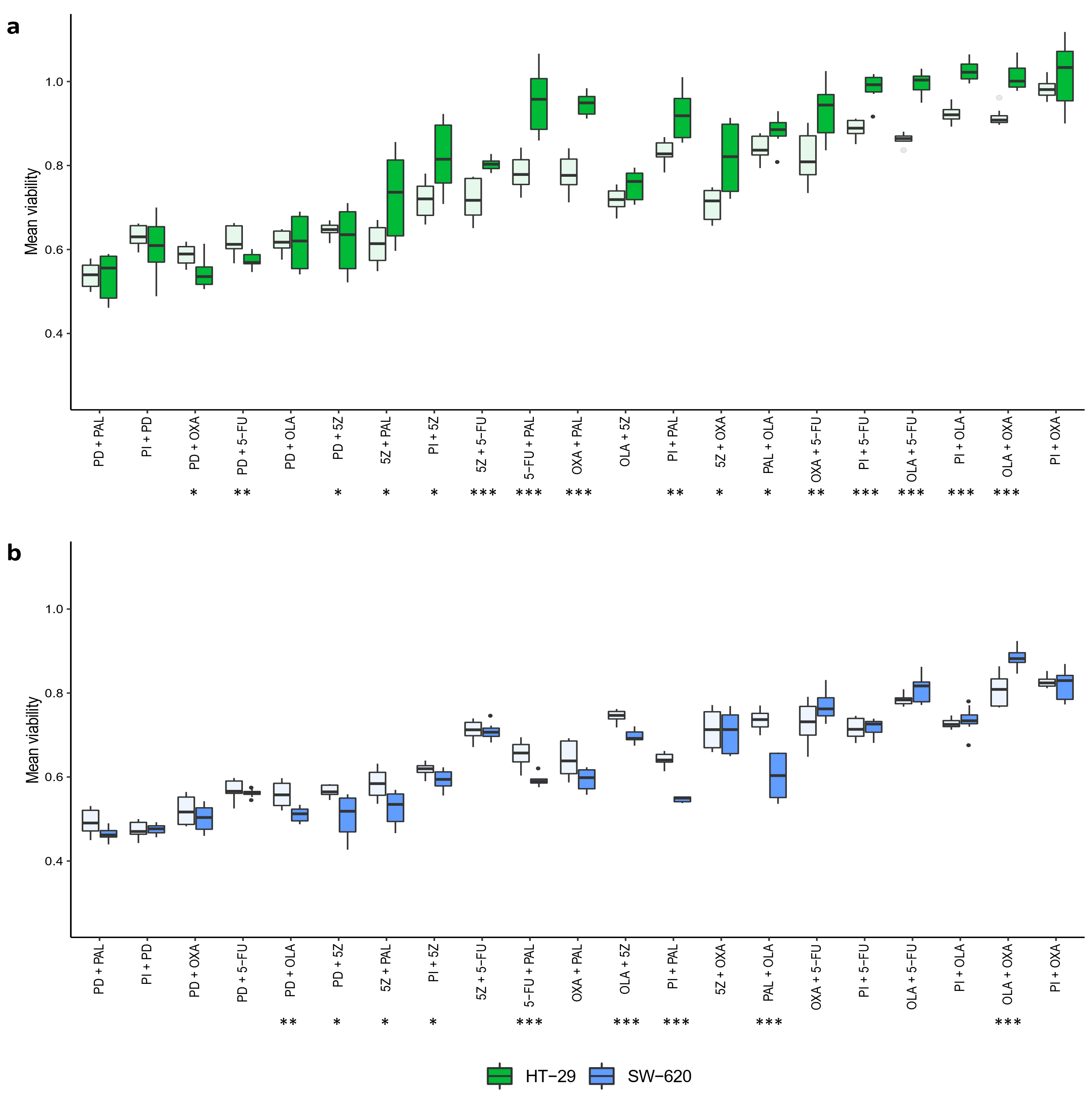


Figure S8 - Average viability in the combination screen (endpoint, 48h). (a) Viability averaged across the matrix per drug combination and culture format (2D = light, 3D = dark) in HT-29, and (b) SW-620 cells. Asterisks (*) indicate statistically significant difference in average viability between 2D and 3D cultured cells per drug combination, with p ≤ 0.05, p ≤ 0.01 and p ≤ 0.001 for *, ** and ***, respectively.


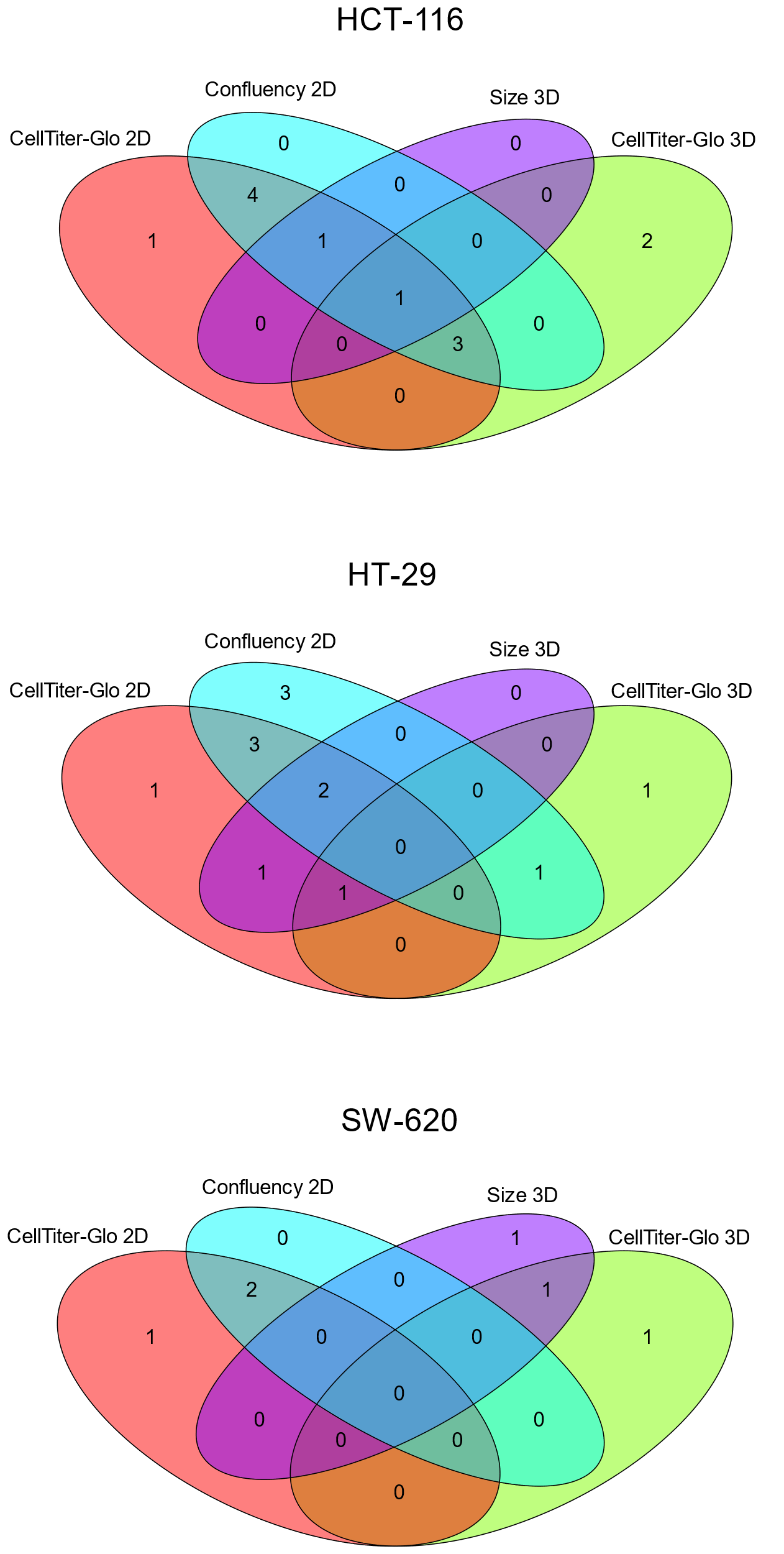


Figure S9 - Venn diagrams showing the number of synergistic drug combinations identified by one or more readouts. Here, synergy is defined as an overall Bliss excess < 0 when data points are averaged over the 5x5 matrix per cell line and drug combination.


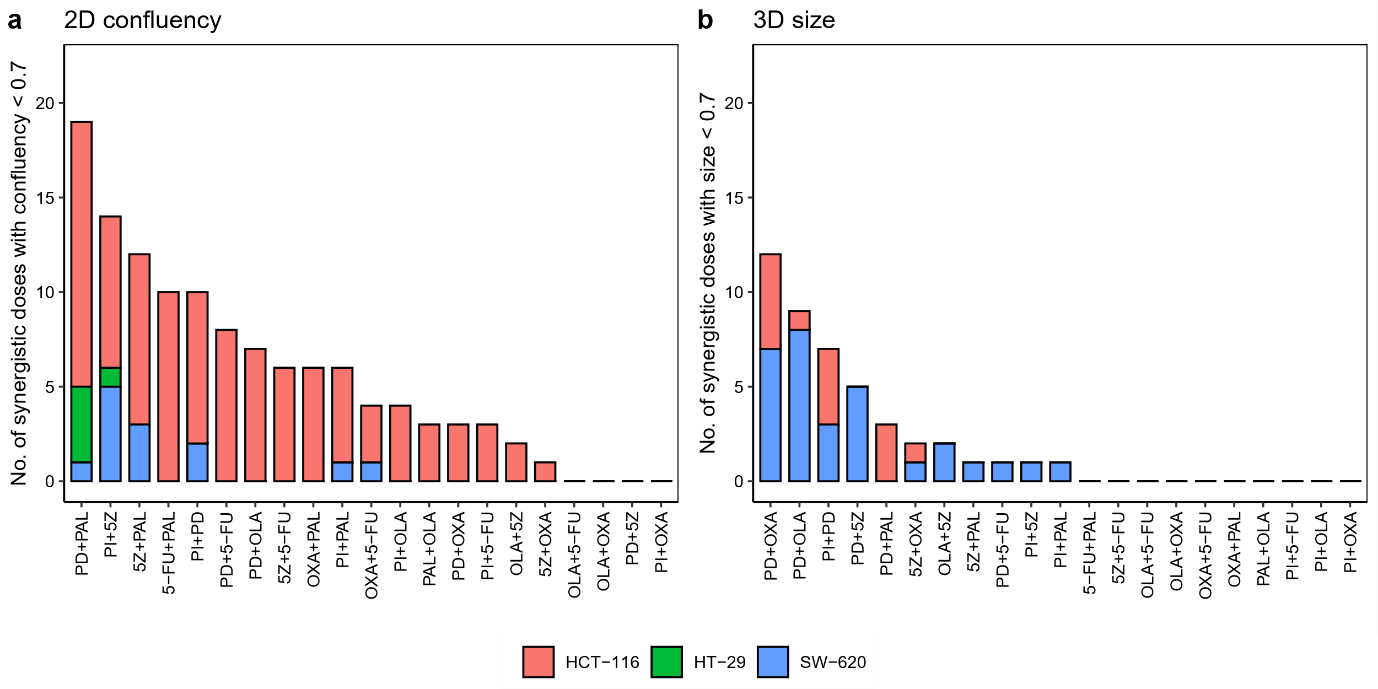


Figure S10 - Number of synergistic doses per combination, cell line and culture format, which reduce confluency or spheroid size to < 0.7. Empty positions along the x-axis indicate combinations for which no doses were observed to fulfil this (alphabetically per culture format).


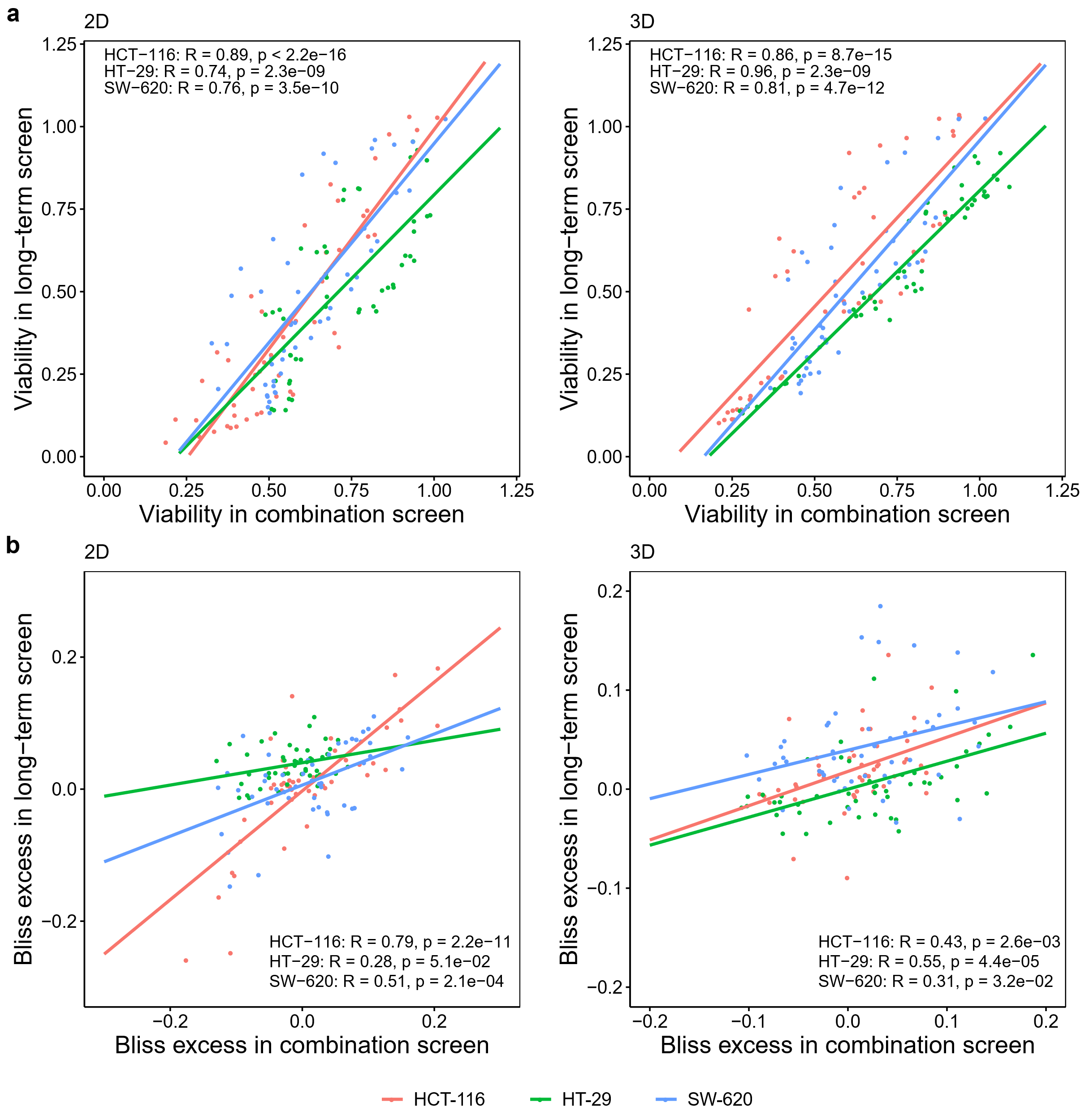


Figure S11 - Combination and 96 hours (here referred to as long-term) screen viability and Bliss excess correlation. (a) Pearson’s correlation between viability at 48h (combination screen) and 96h (long-term screen) in 2D (left) and 3D (right), and (b) Bliss excess scores at 48h (combination screen) and 96h (long-term screen) in 2D (left) and 3D (right).


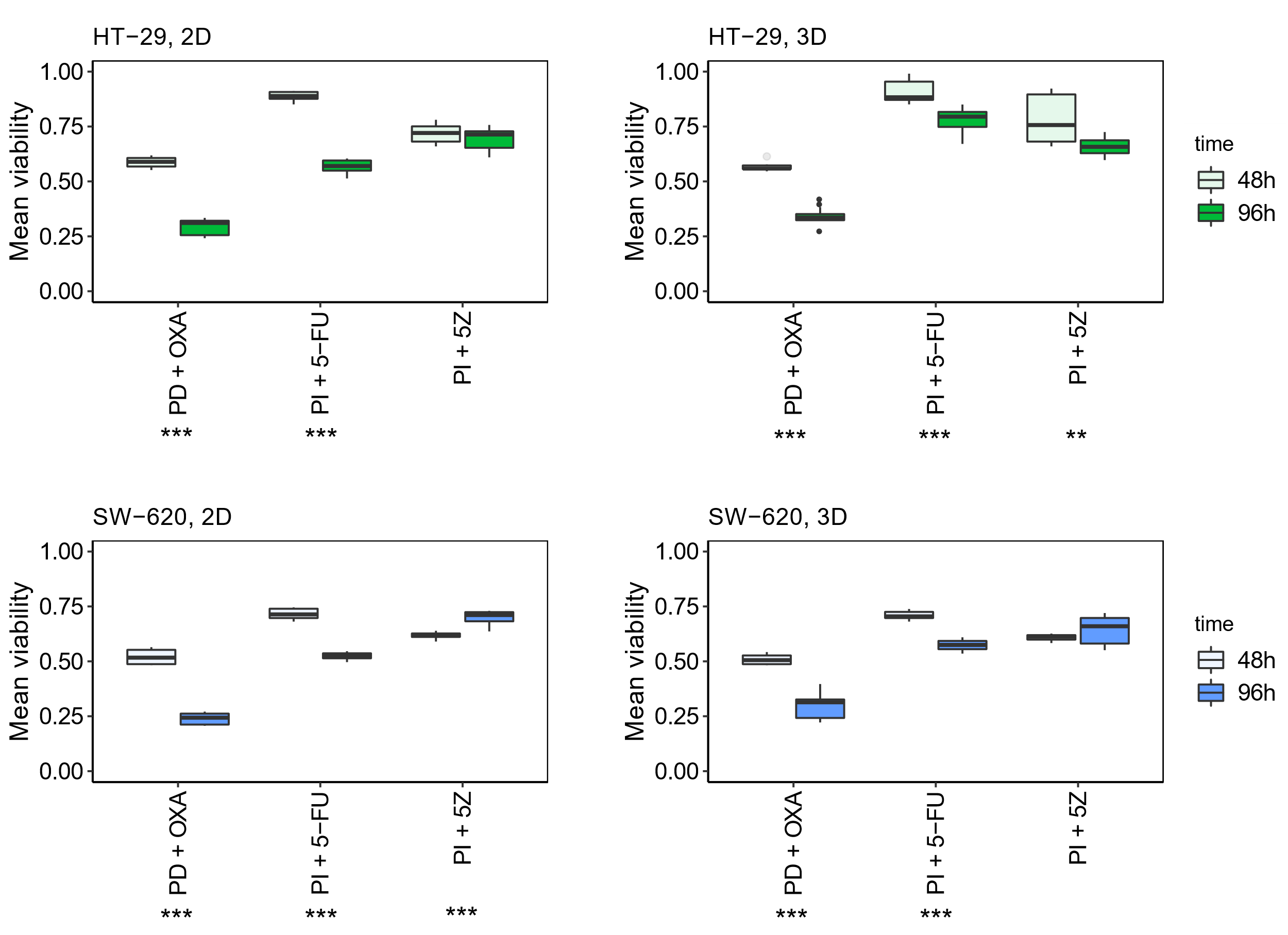


Figure S12 - Average viability per combination in the combination and 96 hours screens. Viability of 2D (left) and 3D (right) cultured HT-29 and SW-620 cells in the combination screen (48h = light) and 96 hours screen (96h = dark). Viability was averaged across the matrix per drug combination, culture format and time-point. Asterisks (*) indicate statistically significant difference in average viability between 48h and 96h per drug combination, with p ≤ 0.05, p ≤ 0.01 and p ≤ 0.001 for *, ** and ***, respectively.


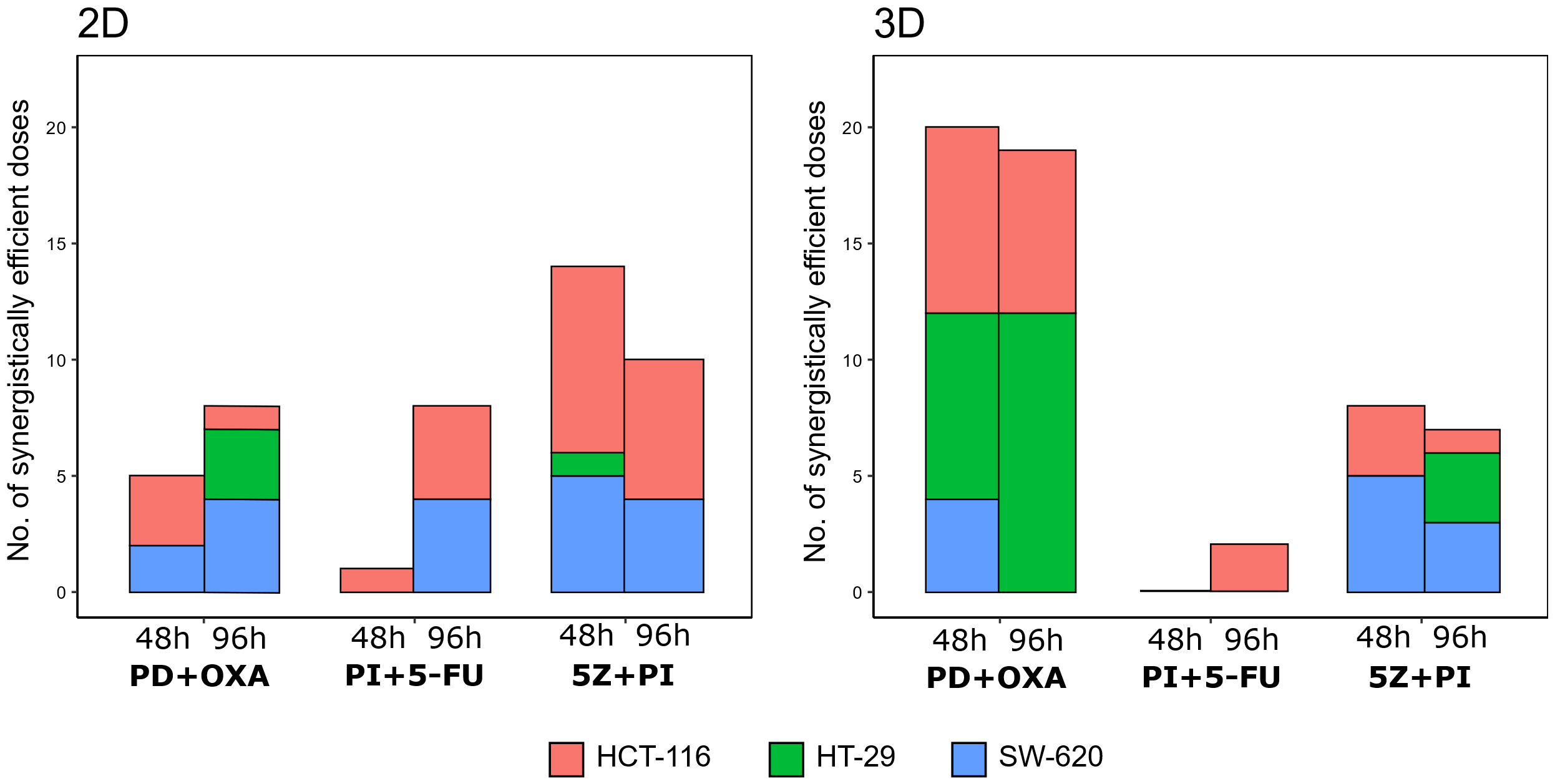


Figure S13 - Number of synergistically effective doses per combination, cell line and culture format at 48h (high-throughput screen) and 96h (96 hours screen).


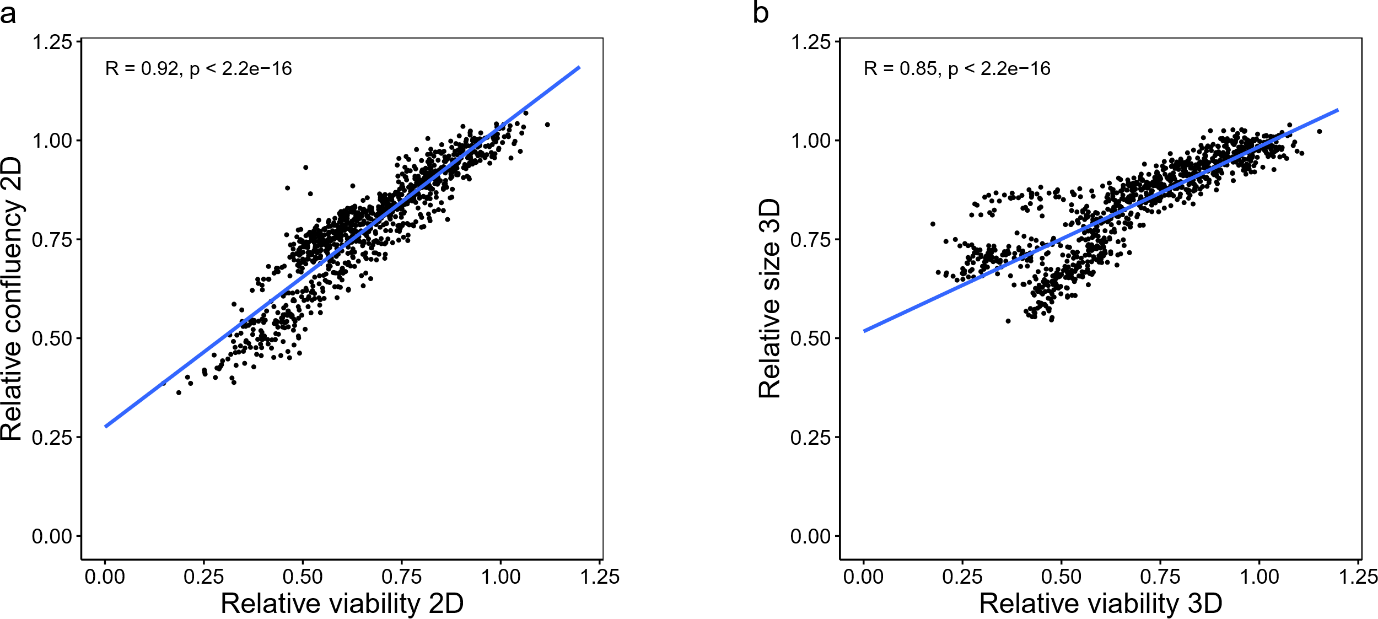


Figure S14 - Correlation between relative readout response in the combination screen in a) 2D, and b) 3D-cultured cells.
